# Supplementary material for: Heterotrimeric G proteins containing Gβ1γ2 subunits exhibit subtype-specific mobility differences in live cells
Source: PLoS One. 2026 Sep 29;21(9):e0359304. doi: 10.1371/journal.pone.0359304 (PMC13623102; doi:10.1371/journal.pone.0359304)
Supplement: Supplementary file 1 [file pone.0359304.s001.pdf]

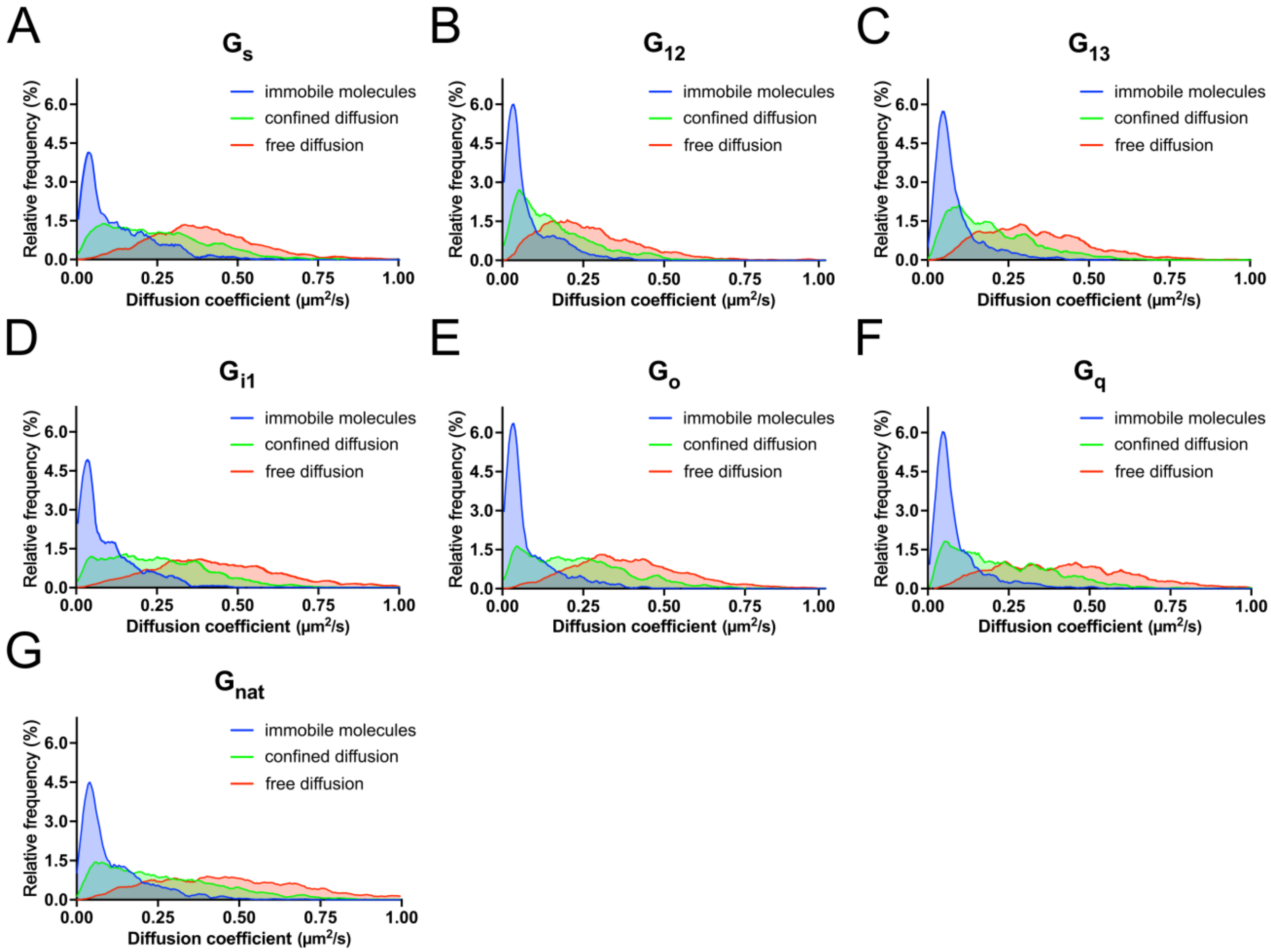

S1 Fig. Distribution of diffusion states in G proteins. Comparison of the distribution of diffusion coefficients of molecular tracks based on diffusion state — immobile (blue), confined (green), freely diffusing (red), in G_s (A), G_{12} (B), G_{13} (C), G_{i1} (D), G_o (E), G_q (F), and G_{nat} (G). Molecule tracks exhibiting directed diffusion are not shown in this representation due to very low sample size.
