# Supplementary material for: Heterotrimeric G proteins containing Gβ1γ2 subunits exhibit subtype-specific mobility differences in live cells
Source: PLoS One. 2026 Sep 29;21(9):e0359304. doi: 10.1371/journal.pone.0359304 (PMC13623102; doi:10.1371/journal.pone.0359304)
Supplement: Supplementary file 2 [file pone.0359304.s002.pdf]

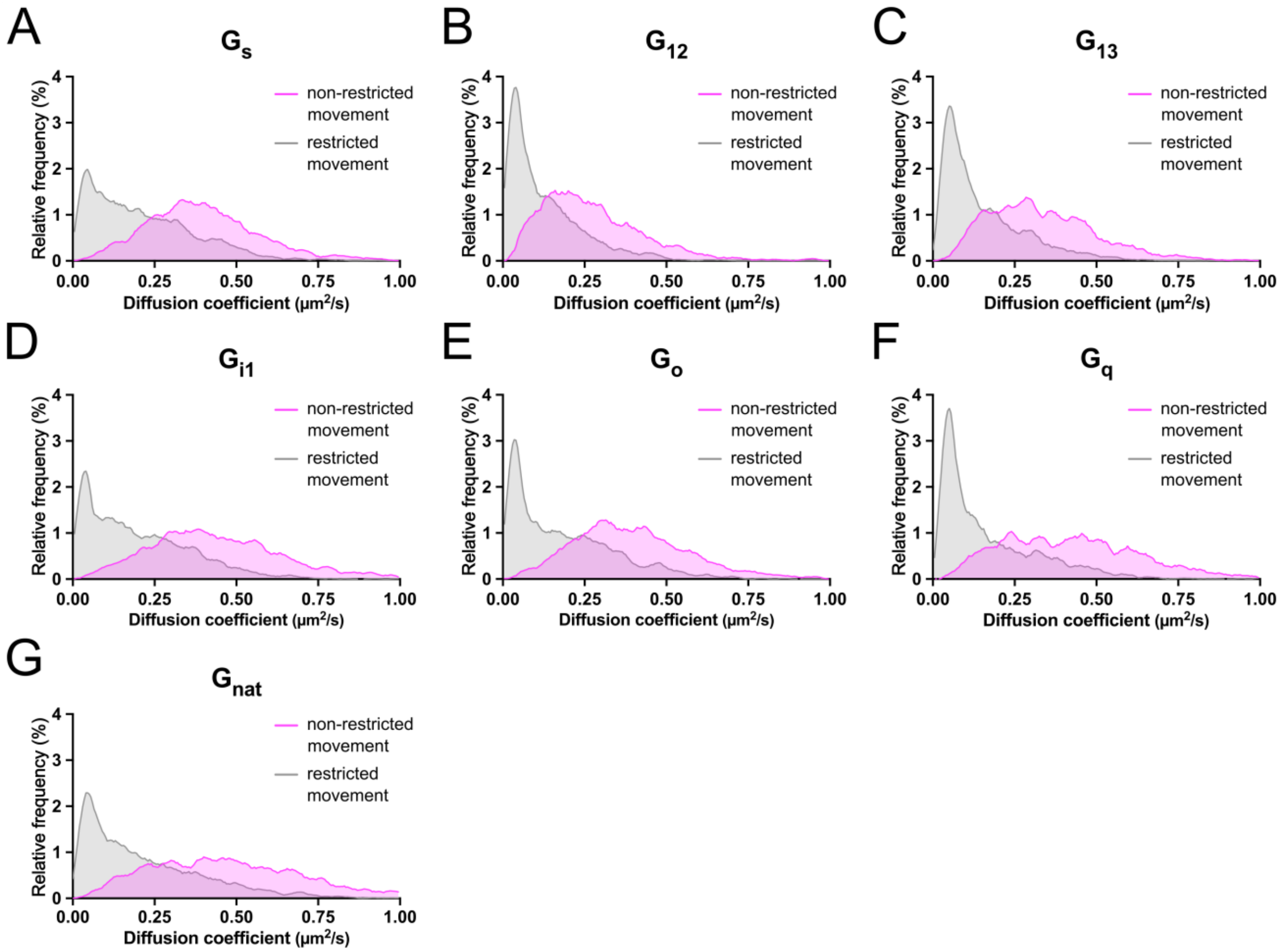

S2 Fig. Distribution of restricted and non-restricted diffusion types in G proteins. Comparison of the distribution of diffusion coefficients of restricted (grey) and non-restricted molecule tracks (magenta) of G_s (A), G_{12} (B), G_{13} (C), G_{i1} (D), G_o (E), G_q (F), and G_{nat} (G).
