# Supplementary material for: Heterotrimeric G proteins containing Gβ1γ2 subunits exhibit subtype-specific mobility differences in live cells
Source: PLoS One. 2026 Sep 29;21(9):e0359304. doi: 10.1371/journal.pone.0359304 (PMC13623102; doi:10.1371/journal.pone.0359304)
Supplement: Supplementary file 3 [file pone.0359304.s003.pdf]

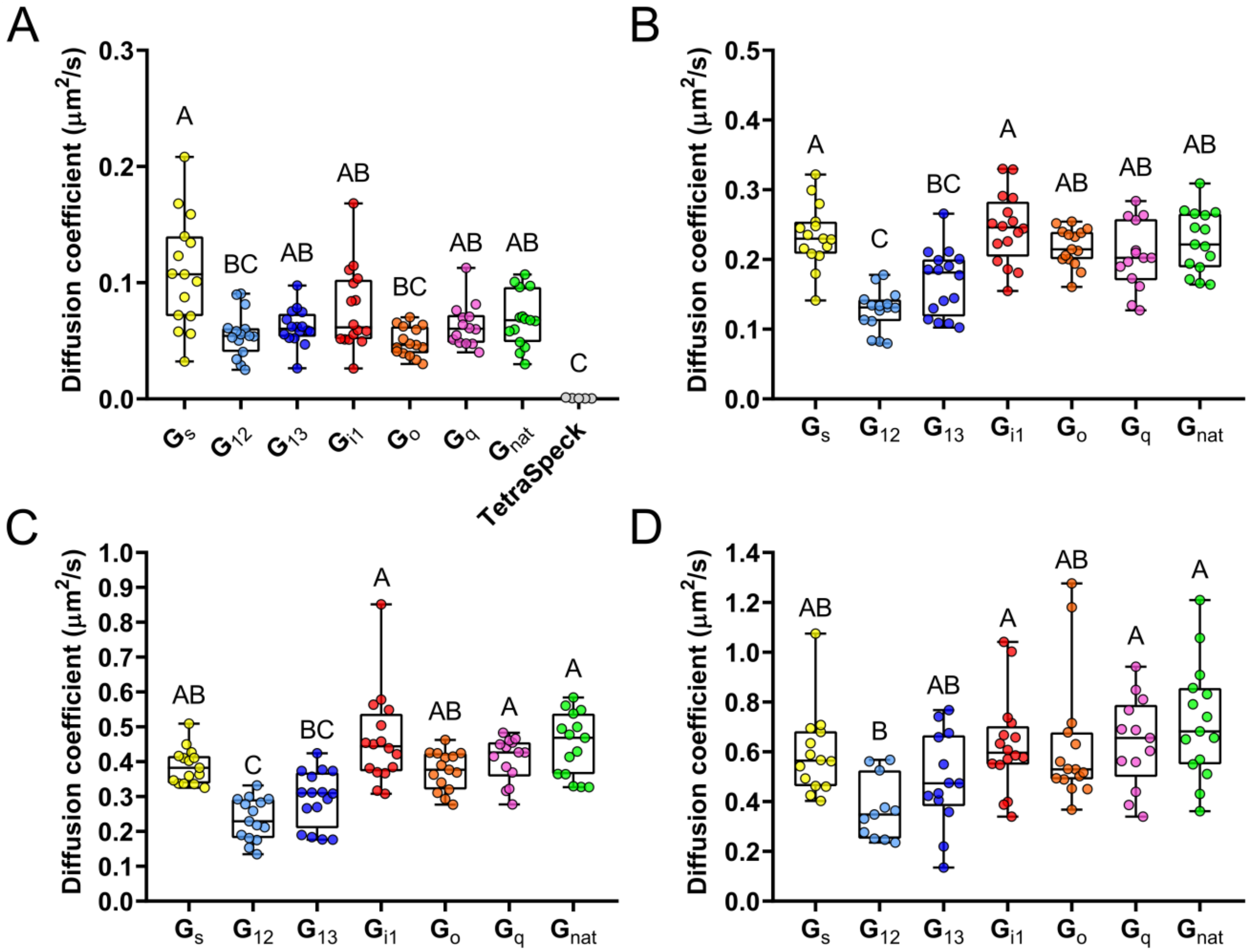

S3 Fig. G proteins show diffusion state-specific differences in mobility. A-D) Box plots of median diffusion coefficients of immobile molecules (A) and molecule tracks exhibiting confined (B), free (C), and directed diffusion (D). Compact letter display above individual datasets indicates similarity grouping based on statistical analysis.
