# Supplementary material for: Heterotrimeric G proteins containing Gβ1γ2 subunits exhibit subtype-specific mobility differences in live cells
Source: PLoS One. 2026 Sep 29;21(9):e0359304. doi: 10.1371/journal.pone.0359304 (PMC13623102; doi:10.1371/journal.pone.0359304)
Supplement: Supplementary file 4 [file pone.0359304.s004.pdf]

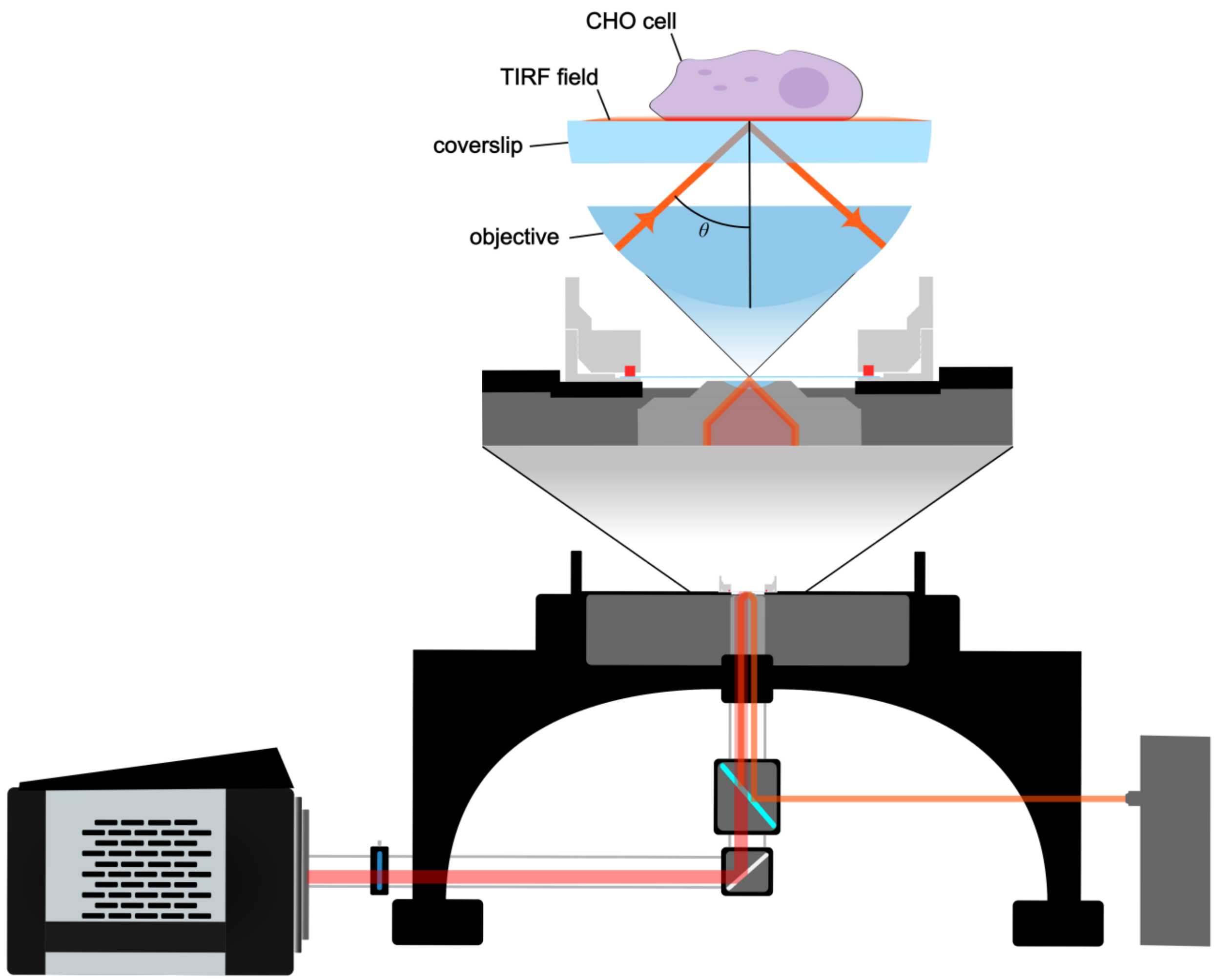

S4 Fig. Single-molecule TIRF microscopy setup. An inverted custom-built TIRF microscope used for single-molecule imaging utilizing a 60x 1.49NA objective lens, a 640 nm laser, and an sCMOS Sona camera. The top diagram shows a close-up of the sample illumination achieved during TIRF microscopy.
