# Supplementary material for: Heterotrimeric G proteins containing Gβ1γ2 subunits exhibit subtype-specific mobility differences in live cells
Source: PLoS One. 2026 Sep 29;21(9):e0359304. doi: 10.1371/journal.pone.0359304 (PMC13623102; doi:10.1371/journal.pone.0359304)
Supplement: Supplementary file 5 [file pone.0359304.s005.pdf]

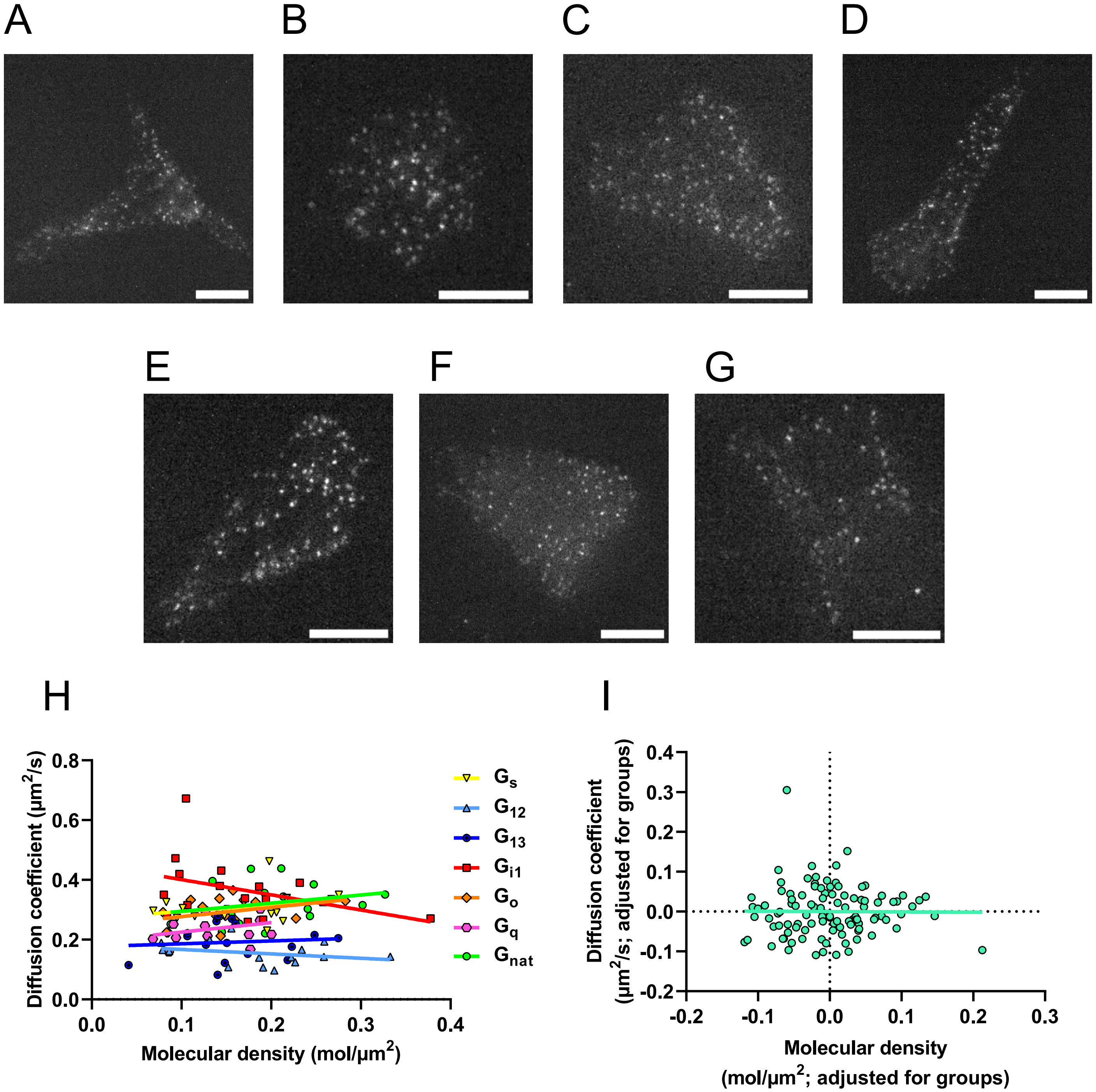
A
B
C
D
E
F
G
H
I
Diffusion coefficient (μm²/s)
Molecular density (mol/μm²)
Gs
G12
G13
Gi1
Go
Gq
Gnat
Diffusion coefficient (μm²/s; adjusted for groups)
Molecular density (mol/μm²; adjusted for groups)
