# Supplementary material for: Heterotrimeric G proteins containing Gβ1γ2 subunits exhibit subtype-specific mobility differences in live cells
Source: PLoS One. 2026 Sep 29;21(9):e0359304. doi: 10.1371/journal.pone.0359304 (PMC13623102; doi:10.1371/journal.pone.0359304)
Supplement: Supplementary file 6 [file pone.0359304.s006.pdf]

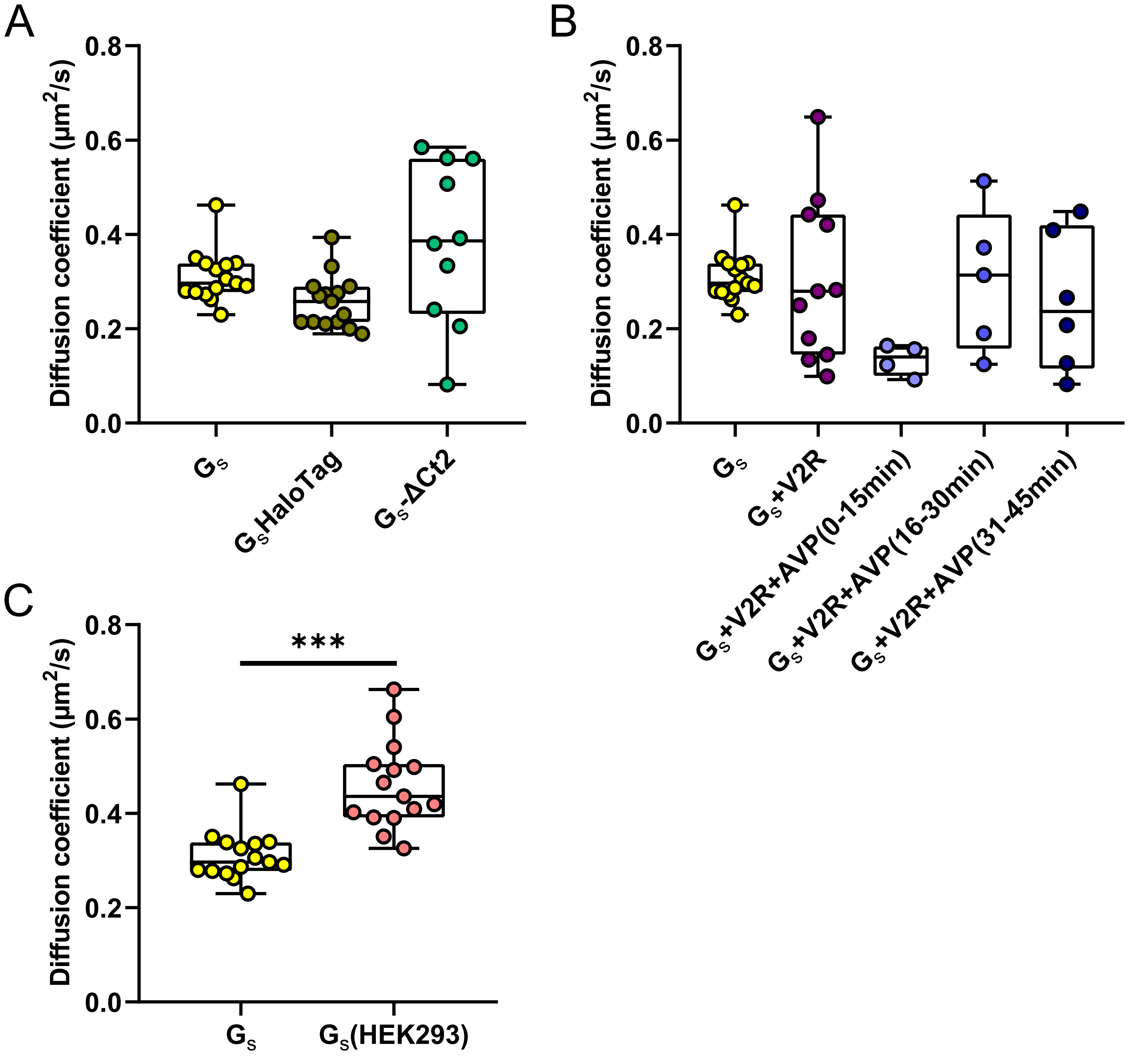
A
B
C
Diffusion coefficient (μm²/s)
0.0
0.2
0.4
0.6
0.8
Gs
GsHaloTag
Gs-ΔCt2
Gs+V2R
Gs+V2R+AVP(0-15min)
Gs+V2R+AVP(16-30min)
Gs+V2R+AVP(31-45min)
***
Gs(HEK293)
