# Supplementary material for: Heterotrimeric G proteins containing Gβ1γ2 subunits exhibit subtype-specific mobility differences in live cells
Source: PLoS One. 2026 Sep 29;21(9):e0359304. doi: 10.1371/journal.pone.0359304 (PMC13623102; doi:10.1371/journal.pone.0359304)
Supplement: Supplementary file 7 [file pone.0359304.s007.pdf]

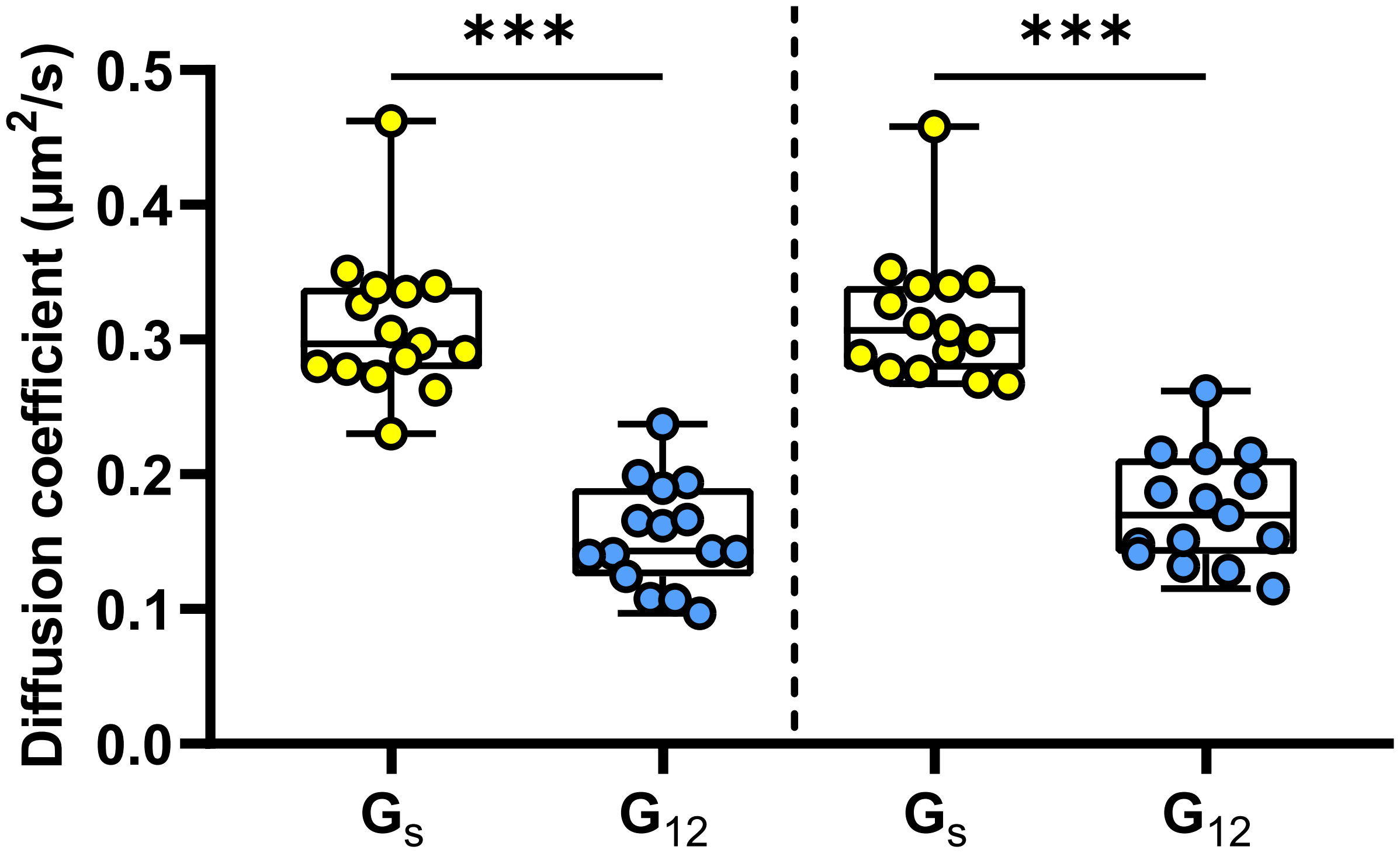

Diffusion coefficient (μm²/s)
0.5
0.4
0.3
0.2
0.1
0.0
***
***
Gs
G12
Gs
G12
