# Supplementary material for: Heterotrimeric G proteins containing Gβ1γ2 subunits exhibit subtype-specific mobility differences in live cells
Source: PLoS One. 2026 Sep 29;21(9):e0359304. doi: 10.1371/journal.pone.0359304 (PMC13623102; doi:10.1371/journal.pone.0359304)
Supplement: Supplementary file 8 [file pone.0359304.s008.pdf]

	**G_s**	**G_{12}**	**G_{13}**	**G_{i1}**	**G_o**	**G_q**	**G_{nat}**
Molecular density (Mol/um^2) ± SD	0.173 ±0.064	0.187 ± 0.071	0.160 ± 0.056	0.165 ± 0.076	0.149 ± 0.052	0.139 ± 0.041	0.202 ± 0.061

Supplementary Table 1. Membrane density of the studied labeled G proteins
