# Supplementary material for: Heterotrimeric G proteins containing Gβ1γ2 subunits exhibit subtype-specific mobility differences in live cells
Source: PLoS One. 2026 Sep 29;21(9):e0359304. doi: 10.1371/journal.pone.0359304 (PMC13623102; doi:10.1371/journal.pone.0359304)
Supplement: Supplementary file 9 [file pone.0359304.s013.docx]

**Reviewer Report**

**PONE-D-26-18579**

**“Heterotrimeric G proteins exhibit subtype-specific mobility differences in live cells.”**

**Significance:**

The findings presented in this study by Alexey Bondar and colleagues provide important insight into an underappreciated layer of GPCR signaling regulation by demonstrating that heterotrimeric G proteins exhibit subtype-specific membrane mobility in living cells. While G protein signaling has traditionally been interpreted primarily through receptor coupling efficiency and downstream effector activation, these results support the emerging concept that the spatial and dynamic organization of signaling components within the plasma membrane itself can strongly influence signaling behavior. The markedly reduced mobility observed for Gα12- and Gα13-containing heterotrimers suggests that individual G protein subtypes may occupy distinct membrane environments or engage differently with membrane nanodomains, cytoskeletal structures, receptors, or signaling scaffolds. Such differences could directly affect receptor encounter rates, signaling kinetics, spatial confinement of signaling events, and access to downstream effectors. Importantly, these observations align with growing evidence that G proteins are highly dynamic signaling entities capable of membrane redistribution, compartmentalized signaling, and subtype-dependent trafficking behavior. Collectively, this work expands the current understanding of G Protein signaling beyond classical conformational activation models and highlights membrane mobility as a potentially important determinant of signaling specificity and cellular response dynamics. However, the manuscript lacks some significant information and contains contradictory facts compared with the already available data. The study is impactful and can be considered for publication in PLOS One after addressing the following concerns.

**Major Concerns**

1) Prior work shows that G proteins are not simply uniformly distributed cytosolic/membrane switches; they undergo subtype- and subunit-dependent membrane association, receptor-dependent immobilization, and activation-dependent redistribution. For example, Gβγ translocation kinetics vary markedly by γ-subtype, and βγ translocation to internal membranes can regulate downstream signaling, including Golgi-localized MAPK pathways. The authors have failed to gather relevant prior information published regarding G protein mobility in the manuscript. G protein mobility is a little old concept, and N. Gautam is one of the pioneers who worked on this. The authors would definitely benefit from reading those manuscripts.
2) This is the most important interpretive limitation. The authors infer that the Gα subunit defines heterotrimer mobility, yet the measured particle is Gγ2-Halo. This inference is acceptable only if the authors demonstrate that most labeled Gγ2-Halo is incorporated into heterotrimers with the intended Gα rather than as free Gβγ, endogenous Gα complexes, or misassembled complexes. The “Gnat” control is useful but insufficient. They should include biochemical or imaging validation: co-expression dependence, membrane recruitment/retention, co-IP, BRET/FRET, or, at a minimum, functional receptor activation controls for representative Gα families. Additionally, multiple prior manuscripts have examined the mobility of different Gγ subtypes.
3) Non-tagged Gα and Gβ1 are CMV-driven, whereas Gγ2-Halo is minP-driven. This design aids sparse imaging but may produce nonphysiological Gα/Gβ/Gγ ratios. G proteins are highly stoichiometry-sensitive, and excess Gα or Gβγ can alter membrane occupancy, nanodomain partitioning, and trafficking. The manuscript needs quantitative expression controls, ideally showing comparable Gα/Gβ levels across conditions or at least normalizing membrane density beyond Gγ2-Halo density. If they are expressing heterotrimers, they need to use a polycistronic plasmid so the ratios match.
4) Gγ identity is a major determinant of Gβγ membrane affinity and translocation kinetics. Prior studies show that the Gγ subtype can strongly regulate βγ dissociation and translocation behavior after GPCR activation. Therefore, the conclusion should be narrowed: “Gα subtype alters the mobility of Gβ1γ2-containing heterotrimers,” not “heterotrimeric G proteins” broadly. Testing at least one additional Gγ subtype, even in a limited comparison such as Gγ7 or Gγ9 with Gαs versus Gα13, would substantially strengthen the paper. Rather than comparing a single Gγ type, it would be essential to examine mobility across Gγ subtypes, including fast, moderate, and slow. The authors should definitely think about changing the title
5) The introduction discusses membrane domains, but the discussion should better integrate the literature showing that Gα and Gβγ can shuttle between the plasma membrane, Golgi, ER, and endosomes, and that βγ translocation can be a signaling mechanism rather than only a localization artifact. This is directly relevant because reduced apparent TIRF mobility could reflect altered residence in PM nanodomains, receptor/effector complexes, or cytoskeletal corrals rather than intrinsic “slower heterotrimer diffusion.” Authors should remember that, not only Gα but also Gγ subtypes have lipid anchors that keep them on membranes. Gγ subtypes preferentially harbor two lipid anchor types, which facilitate membrane residence.
6) The data indicate that several tracking parameters were adjusted “case-by-case,” including the quality threshold, linking distance, and gap-closing distance. This is a potential source of bias because slower and faster particles can be differentially affected by linking criteria. The authors should provide either fixed final parameters per condition, sensitivity analyses, or a reanalysis using identical parameters across all groups.
7) The figures appear to pool molecule tracks, but tracks from the same cell are not independent biological replicates. The authors mention extracting median values and reporting population sizes for each cell, yet Figure 3A appears to plot cell-level medians, whereas Figure 3B pools tracks. The authors should clearly state the unit of analysis: molecule track, cell, imaging day, or independent transfection. Statistical testing should be performed on biological/cell-level summary metrics, not on pooled tracks.
8) The claim that restricted Gα12/13 movement “likely points to physiological interactions with other signaling cascade members and regulators” is plausible but not proven. Alternative explanations include altered membrane lipid affinity, palmitoylation state, incomplete heterotrimer assembly, expression-level differences, cytoskeletal confinement, Gγ2-dependent behavior, and even endogenous Gγ expression levels. Authors should address these inconsistent and unsupported claims.

**Minor issues**

1) The phrase “unification and amplification of perceived extracellular chemical and physical stimuli” in the abstract is awkward. I would revise to: “Heterotrimeric G proteins transduce and amplify extracellular chemical and physical cues downstream of GPCRs.”
2) “antiobiotic/antimycotic” should be corrected to “antibiotic/antimycotic.”
3) The manuscripts would again benefit from overall grammar, spelling, and wording revision. For example, the conclusion should avoid “point out to” and use “point to.”
