# Supplementary material for: Heterotrimeric G proteins containing Gβ1γ2 subunits exhibit subtype-specific mobility differences in live cells
Source: PLoS One. 2026 Sep 29;21(9):e0359304. doi: 10.1371/journal.pone.0359304 (PMC13623102; doi:10.1371/journal.pone.0359304)
Supplement: Supplementary file 10 [file pone.0359304.s015.docx]

The authors have responded constructively and thoroughly to the previous round of review. The literature framing is much improved, the statistical unit is now better, the unsupported mechanistic claim has been removed, and the new Limitations section is a welcome addition. The new supporting data (S5-S7) address the most important concerns within the scope of this study. I have only a few residual points, most of them textual.

The expanded "G protein localization" and "G protein mobility" sections now situate the work appropriately, and the added references (Gautam and colleagues, Mystek, Abankwa) better reflect the field.

The authors adequately validate that Gγ2-Halo reports the intended heterotrimer, given the scope. The Gαs-HaloTag comparison, the V2R/AVP activation control, and the Gαs-ΔCt2 data collectively support functional assembly. One suggestion to strengthen the argument at no experimental cost: the authors have a strong internal control they do not use. If Gγ2-Halo were failing to incorporate with exogenous Gα12/Gα13, those conditions should converge on the Gnat value (~0.32 µm²/s); instead, they diverge sharply (0.14 and 0.20 µm²/s). This logic directly argues against the misassembly scenario and belongs in the Limitations section.

Promoter mismatch and subunit stoichiometry. Addressed as far as feasible. The reciprocal-promoter experiment in S6A is the appropriate control, and the limitation is now stated plainly.

Narrowing the conclusion/title. The new title and abstract largely address the scope.

The fixed-parameter reanalysis in S7 was needed and has been addressed. It is worth noting in the text that the fixed linking distance (0.7 µm) sits at the low end of the range used, which, if anything, biases against the faster groups.

The explicit statement at the end of the Statistical analysis section, the revised Fig. 3A legend, and the S2 Table resolve the data analysis issue.

The unsupported interpretive claim has been addressed by removing the sentence.

- However, the abstract overstates the comparison involving Gq. It states that Gα12/Gα13-containing heterotrimers show markedly reduced mobility "compared to those containing Gαi/o, Gαs, and Gαq." This is not what the statistics show: in Fig 3A, Gq is assigned BC and shares group C with G12 and G13, and in Fig 4B, Gq groups with G12 and G13 (B). The Results text is appropriately careful ("somewhat in between these groups"), but the abstract is not. Please either remove Gαq from that sentence or qualify it (e.g., "with Gαq intermediate").
- Residual overgeneralization in headings and the conclusion. The title and abstract were narrowed to Gβ1γ2-containing heterotrimers, but the Results heading ("Different families of G proteins exhibit distinct mobility patterns in live cells"), the closing sentence of the Discussion ("strong differences in mobility of different types of heterotrimeric G proteins"), and the phrase "in the broader context of G protein mobility" still generalize beyond the tested pairing. Please align these with the revised title.
- The single Gγ subtype is not listed as a limitation. Given that Gγ identity itself determines Gβγ membrane behavior, the restriction to Gβ1γ2 warrants a fourth item in the Limitations section, alongside expression level, density, and cell type.
- Statistical reporting is incomplete. The Methods define ε² and rank-biserial correlation, but no test statistics, p-values, or effect sizes appear in the Results or in a supporting table (only the regression output is reported). Please report these, state which test was applied to each figure (Kruskal-Wallis/Dunn vs Mann-Whitney), and specify the α and correction used for the compact letter display in Figs 3A and 4B.
- S6 and S7 carry a heavy interpretive burden for the descriptive data. S6A is cited in response to four separate reviewer concerns about expression levels, yet the legend states that no statistical inference was performed due to low sample size. This is understandable, but the wording throughout ("comparable mobility," "no significant effect") should be softened, since the absence of testing is not evidence of equivalence. Reporting n and the observed ranges for each S6 condition would help.
- Clarify the basis for the diffusion-state percentages. The values quoted in the Results (59/60/57% vs 44/41/41%) should be identified as pooled-track fractions or as means/medians of per-cell fractions, consistent with the new statement that cell-level medians are the statistical unit.
- Membrane density is reported as 0.14-0.20 molecules/µm² in the Results but as ~0.1-0.3 mol/um² in the TIRF Methods; please reconcile and use µm² consistently.
- "lipidation motive" 🡪 "lipidation motif" (Discussion).
- Inconsistent naming of the truncation mutant: "Gαs-ΔCt2" vs. "Gs-ΔCt2" (Methods, Limitations, S6 legend).

I think the manuscript is ready for publication after these edits.
