# Supplementary material for: Heterotrimeric G proteins containing Gβ1γ2 subunits exhibit subtype-specific mobility differences in live cells
Source: PLoS One. 2026 Sep 29;21(9):e0359304. doi: 10.1371/journal.pone.0359304 (PMC13623102; doi:10.1371/journal.pone.0359304)
Supplement: Supplementary file 11 [file pone.0359304.s016.docx]

# Reviewer #1

: This manuscript investigates whether the lateral mobility of heterotrimeric G proteins on the plasma membrane differs depending on the type of Gα subunit, using live-cell TIRF single-molecule imaging and single-particle tracking. The authors introduced untagged Gα subunits, Gβ1, and low-expression Gγ2-HaloTag into CHO-K1 cells, and used Gγ2-HaloTag as a common reporter to compare the membrane diffusion of heterotrimeric G proteins presumed to contain each Gα subtype. They report that G proteins containing Gα12 and Gα13 show lower diffusion coefficients than those containing Gαs, Gαi1, and Gαo. They further report that Gα12- and Gα13-containing G proteins have a higher fraction of restricted diffusion components classified as immobile and confined diffusion.
This study may provide useful insight into the spatiotemporal regulation of GPCR–G protein signaling. In particular, the observation that Gα12/Gα13-containing heterotrimers exhibit low mobility is interesting. However, several important points require additional data or clarification to sufficiently support the authors’ claims.

Major comments

1. The relationship to previous studies and the novelty of the present work should be clarified
The central concept of this study is that the membrane mobility of G proteins is heterogeneous and can be altered by subunit composition, membrane domains, and interactions with GPCRs, which has already been demonstrated in previous single-molecule studies. For example, heterogeneous mobility of G proteins, membrane microdomain localization of Gq heterotrimers, changes in membrane localization and diffusion behavior depending on combinations of Gα and Gγ subunits, and reduced G-protein mobility upon interaction with GPCRs have all been reported previously (Oerez JMB 2006; Abankwa J Cell Sci 2007; van Hemert J Cell Sci 2010; Mystek Cells 2019...).
Therefore, the authors should describe the claim that “the type of Gα subunit determines the mobility of heterotrimeric G proteins” not as an entirely new concept, but rather as an extension of previous studies. The specific novelty of the present study appears to lie in the side-by-side comparison of multiple Gα subtypes under the same experimental conditions, particularly in showing that heterotrimers containing Gα12 and Gα13 exhibit low mobility. This point should be clearly stated in the Introduction and Discussion.

**Response:** Thank you for the comment; we modified the manuscript to better reflect the relationship of our results to previously published data. (l. 92-96)

2. It is necessary to validate that Gγ2-HaloTag reflects the mobility of the intended Gα-containing heterotrimers
In this study, the authors use Gγ2-HaloTag as a common reporter and estimate the mobility of heterotrimeric G proteins containing each Gα subtype by co-expressing the respective Gα subunits. However, it has not been sufficiently demonstrated that Gγ2-HaloTag preferentially forms heterotrimers with the intended exogenous Gα subunits.
Gγ2-HaloTag may associate with endogenous Gα subunits or may exist as free Gβγ. Therefore, it is unclear whether the observed differences in mobility reflect differences among heterotrimers containing each Gα subtype, or instead reflect differences in association efficiency with exogenous Gα, competition with endogenous Gα, or the proportion of free Gβγ.
The authors should demonstrate, by co-immunoprecipitation, membrane localization analysis, expression-level comparison, functional complementation, or another independent approach, that Gγ2-HaloTag is an appropriate reporter for heterotrimers containing the intended Gα subunits. At minimum, the authors should provide representative imaging examples for cells expressing each Gα subunit and clearly discuss this point as a limitation of the study.

**Response:** We thank the reviewer for their recommendation of relevant controls. We, as well as others, have previously shown that Gα_s_ [1], Gα_i1_ [2, 3, 4, 5], and Gα_o_ [2, 6] subunits form functional heterotrimers with Gβ_1_γ_2_ dimers and can be activated by GPCRs. Though we could not carry out all of the proposed experiments, we included additional supporting information in Supporting Figures 5 and 6. These include, as suggested, images of cells under each condition, as well as a mobility comparison between G_s_ protein labeled at the Gγ subunit and directly at the Gα_s_ subunit. The data show that both labeling approaches produce the same heterotrimer mobility outcomes. Additionally, the data in Supporting Figure 6B showcase the decrease in the mobility of G_s_ containing labeled Gγ_2_-HaloTag in the presence of an activated V2R receptor, consistent with previously shown activation of a functional G_s_ heterotrimer [1]. This suggests that overexpressed Gα_s_ forms functional heterotrimers with Gβ_1_ and Gγ_2_-HaloTag, indicating that the observed high values of the diffusion coefficient are not caused by reduced binding of Gγ_2_-HaloTag with certain overexpressed Gα subunits, such as Gα_s_. We were not able to create HaloTag versions of other Gα subunits because direct labeling of Gα subunits can affect their functional activity [5, 2], and newly created constructs require extensive biochemical characterization. Therefore, direct testing of other labeled Gα subunits remains beyond the scope of this study. We have also added a Limitations section to the manuscript. (l. 246-271)

3. The effects of expression levels and membrane molecular density should be evaluated more rigorously
The authors state that Gγ2-HaloTag was used under low-expression conditions, but the explanation is insufficient regarding how the labeled molecule density, Gα subunit expression level, and cell-to-cell variability in each condition may affect the diffusion coefficients. Differences in expression level or membrane density may influence molecular crowding on the membrane, the stoichiometry of Gβγ and Gα, competition with endogenous G proteins, and the fraction of free Gβγ.
The membrane density of Gγ2-HaloTag in each condition should be clearly shown in the main text or in a major supplementary figure, and the authors should demonstrate that there is no correlation between membrane density and diffusion coefficient. It is also necessary to show that the expression levels of the different Gα subunits are comparable, or at least within a range that does not substantially affect the interpretation of the results. In particular, because the Gα13-IRES-mCherry and Gαq-IRES-mCherry constructs differ from the other pcDNA3.1 expression constructs, the authors should explain the expression levels and criteria used for cell selection.

**Response:** The manuscript was expanded by a linear regression analysis of membrane density and diffusion coefficient values (S5I Fig), showing no correlation between the two. Though, as correctly pointed out by the reviewer, this approach is limited as it only incorporates the membrane density of Gγ_2_-HaloTag, and not the overexpressed Gα and β. This weakness is, in part, offset by the data showcased in Supporting Figure 6A, showing that G_s_ heterotrimers containing a HaloTag-labeled Gα_s_ subunit expressed under the minP promoter exhibit similar mobility to those with an overexpressed unlabeled Gα subunit. This suggests that the difference in expression levels of both Gα and Gγ does not have a critical impact on the observed mobility. At the same time, if we assume expression level differences as the main driver of the decreased mobility in G_12_ and G_13_ through effects such as membrane crowding, that would necessitate strongly and consistently increased expression levels of Gα_12_ and Gα_13_ compared to the other subunits, which we are unaware of there being a precedent for. At the same time, we are aware of the limitations of this approach, and we now explicitly acknowledge it in the manuscript. (l.246-266)

Cell selection was based primarily on single-molecule analysis suitability, specifically, the lack of non-specific fluorescence and Gγ_2_-HaloTag membrane density, as well as healthy cell appearance. We made sure to include this information in the manuscript as well. (l. 318-319)

4. The statistical unit and sample size should be clarified
In the present manuscript, it is not sufficiently clear whether the statistical analyses were performed based on individual tracks or on summary statistics such as median values per cell.
The authors should clearly state what each point in each figure represents and whether the unit of statistical testing was the track, the cell, or the independent experiment. Ideally, statistical analyses should be performed using cell-level values, and the numbers of independent transfection experiments, analyzed cells, and tracks should be clearly indicated for each condition.
**Response:** Thank you; the number of experiments, analyzed cells, and tracks has been included in Supporting Table 2, and the manuscript has been adjusted accordingly. (l. 141-148, 390-392, 518-526). We agree that only individual cells can serve as biological replicates and would like to emphasize that all statistical analyses were performed using cell-level values.

Minor comments

1. Since Gα itself was not directly labeled in this study, expressions such as “Gα12 mobility” should be avoided. The measured target should be described more precisely, for example as “the mobility of Gα12-containing heterotrimers inferred from Gγ2-HaloTag tracking.”

**Response:** We thank the reviewer for the suggestion; we revised our manuscript accordingly. (l. 144, 208, 247-248)

2. In Fig. 4, the authors should indicate whether statistical comparisons were performed for the proportions of restricted and non-restricted movement.

**Response:** We have updated Fig 4 to better showcase our results and allow for robust statistical analysis. (l. 167-175)

3. In the Methods, the authors state that fixed parameters were used, whereas they also state that the detection threshold, linking distance, and gap-closing distance in the TrackMate analysis were adjusted for each sample. The authors should clarify which parameters were fixed and which were optimized for each condition.

**Response:** We thank the reviewer for the insightful comments; we revised the manuscript to improve clarity and better explain our approach. (l. 115, 336-337)

4. The term “antiobiotic/antimycotic” in the Methods appears to be a typographical error for “antibiotic/antimycotic” and should be corrected.

**Response:** Thank you, we updated the manuscript accordingly. (l. 282)

# Reviewer #2:

In the very broad and important field of cellular signalling, G protein cascades have exceptional significance not only because they are involved in nearly all physiological processes in humans, but also because they are among the most widely targeted pathways in therapeutic interventions. Therefore, the manuscript by Kuchynka and collaborators may potentially arouse interest among a wide audience. While the authors present a bunch of interesting and well elaborated data, several important methodological limitations significantly weaken the support of the conclusions drawn. As a result, the whole story is too preliminary to be publishable in its current form, and the authors should consider a few issues listed below.
- First of all, the levels of overexpressed non-tagged G proteins within target cells are to large extent unknown. In fact, the authors provide membrane densities in Table S1, but this is most probably based on the signal from G gamma subunit. Unfortunately, the methodology is not presented, and the table description is misleading since the specified G proteins were not directly labelled. Apparently, the real stoichiometry of all the G protein subunits within cells was not taken into consideration, although it may severely influence behaviour of these proteins (i.e. oligomerization, diffusion, membrane affinities, etc.).
**Response:** We highly appreciate the comment. Indeed, expression levels of the proteins of interest are important for the interpretation of the results. We have now performed additional experiments (S6 Fig) and show that direct labeling of the Gα_s_ subunit yields diffusion coefficients comparable to those determined through Gγ_2_ labeling. Since this combination of Gα_s_ with Gβ_1_γ_2_ dimers yields higher diffusion coefficient values than observed for the Gα_12_ subunit, we believe that our results can’t be explained solely by expression level differences. Additionally, we have now provided representative images of cells with all construct combinations and evaluated the dependence of Gγ_2_ density and diffusion coefficient. We have found that Gγ_2_ density does not directly correlate with diffusion coefficient values. At the same time, we are aware of the limitations of our approach and have now explicitly indicated this, as well as our methodology behind membrane density measurements in the text of the manuscript. (l. 246-266, 559-561)

- As the authors aptly point out in the discussion, it should be expected that the diffusion of G proteins strongly depends on the level of their lipidation. It is already known that overexpression of proteins in mammalian cells may overload the intracellular enzymatic system responsible for lipidation/delipidation and thus result in partial lipidation (or artefactual change in lipidation pattern) of the proteins of interest (see e.g. 10.3390/membranes11090715). Thus, without experimental confirmation (e.g. via “acyl-RAC” method, see DOI: 10.1194/jlr.D011106) that the overexpressed G proteins exhibit posttranslational modifications patterns identical to physiologically relevant counterparts the data presented within the manuscript would have very limited significance.
**Response:** Thank you for the comment. After reviewing the presented literature, we have decided to include partial lipidation as a possible element of variability in the Discussion section of the manuscript. At the same time, we believe that lipidation alone cannot explain the variability observed in our results for a few reasons. Partial lipidation would likely result in subpopulations of G proteins with distinct mobility patterns, especially so in Gα_12,_ which only contains a single palmitoylation site, meaning it can only exist within palmitoylated and non-palmitoylated states. Broadly speaking, partial lipidation of subunits should lead to a decrease in membrane affinity and an increase in lateral membrane mobility. This would point to our results showing not decreased mobility in G_12_, but increased mobility in all the other subunits. This is possible, but the differences in lipidation would likely produce vastly different outcomes in myristoylated subunits Gα_i1_ and Gα_o_, in which myristoylation is mandatory for proper trafficking and membrane attachment, compared to subunits that are only palmitoylated, such as Gα_s_ and Gα_q_. Another reason why we believe that lipidation variance related to overexpression is not a major driver of the observed mobility differences is that G_s_ heterotrimers exhibit comparable mobility in the presence of both overexpressed and underexpressed Gα_s_ (S6A Fig). (l. 221-223)

- To further clarify the importance of individual structural elements of G proteins (as discussed within l. 177-184 and l.194-206), site-directed mutagenesis could be performed in order to generate proteins that lack particular functionalities.
**Response:** Thank you for the thoughtful suggestion. While unable to carry out the proposed steps, in Supporting Figure 6A, we have included data from heterotrimers containing a Gα_s_ subunit mutant lacking two C-terminal amino acids. Our data suggest its mobility might be slightly elevated compared to regular G_s_ heterotrimers, which could correlate with reduced efficiency in protein-protein interactions, particularly GPCR binding, due to the truncated C-terminus. Other mutant variants of various subunits would be an interesting direction for future research. (l. 257-260)

- Would the behaviour of G proteins vary depending on host cell type? The presented data were generated using only one cell line and thus raise a question whether the observed effects are cell-specific.

**Response:** We have added mobility measurements of the G_s_ protein from the HEK-293 cell line in Supporting Figure 6C. These data suggest a significant difference in the mobility between the two cell lines and point to an increased importance of cell line identity on the overall mobility of membrane proteins. We additionally discuss this as a possible point of limitation in the manuscript and argue that absolute values of diffusion coefficients from experimental systems intrinsically have limited value, but the differences between them point to important trends in mobility of G protein heterotrimers. (l. 267-271)
- Minor issues include: the colour code for G protein heterotrimer models on Figs 1 and 2 should be defined; caveolae have biochemical features of lipid rafts and thus should be considered as specialized subset of lipid rafts (see e. g. DOI: 10.1038/nrc3915 and refer it to l.41-48 of the main text); the panel B of Figure 4 is redundant – using two distinguishable patterns (on top of the already used colour code) on the panel A would do a better job; referring to more recent papers in the field of G protein raft partitioning is highly advisable (e.g. DOI: 10.3390/biomedicines11020557, DOI: 10.1007/978-3-540-72843-6, to name a few).

**Response:** We thank the reviewer for the insightful comments; we revised the manuscript to improve clarity and figure presentation. (l. 70,119-120; 46-58, 65; 169-175; 32-58)

# Reviewer #3

: The authors report G protein subfamily-specific differences in lateral membrane mobility, with Gα12- and Gα13-containing heterotrimers displaying particularly slow diffusion. The manuscript is concise, clearly written, and supported by well-designed figures. However, the current study remains rather preliminary and would benefit from additional experimental support and/or a more cautious interpretation of the data.

1. Results (lines 84–86): The authors state that direct labeling of Gα subunits can affect their interaction with Gβγ, and therefore use labeled Gγ2 as a universal mobility readout. It would be important to clarify whether labeling of Gγ might itself influence Gα–Gβγ interactions. Even if Gγ is uniformly labeled, it remains possible that such labeling differentially affects distinct Gα subunits. In addition, does Gα mobility change depending on whether Gγ is labeled or not? These points should be addressed experimentally or, at minimum, discussed as important caveats.
**Response:** Thank you for the comment. Fluorescent labeling can indeed cause various issues with protein interaction, folding, or steric hindrance, meaning reporter design inextricably comes with a certain degree of invasiveness. Our labeling approach has been focused on minimizing this degree of invasiveness by inserting the fluorescent protein at the N-terminus of the Gγ subunit, which is the furthest-facing part of the heterotrimer from both the membrane and the receptor interaction centers, such as the Gα C-terminus. At the same time, using a single labeling approach across experiments avoids the introduction of additional variability that comes with using multiple different insertion sites. Despite that, this approach still carries a risk of functional interference. Because of that, we have included a mobility comparison between G_s_ heterotrimers labeled at the Gγ subunit and the Gα subunit (S6A Fig). The data suggest that the mobility of Gα_s_-labeled heterotrimers does not significantly differ from the G_s_ mobility observed in our study. (l. 252-254)

2. Figure 3A: To what extent could the slower diffusion of Gα12- and Gα13-containing complexes be related to differences in expression levels or abundance? How comparable are the expression levels of the various Gα proteins, and how does altered expression of individual subunits affect mobility? These are critical aspects that should be clarified before drawing conclusions about intrinsic subtype-specific mobility.

**Response:** We thank the reviewer for the comment. Expression level differences are an important variable when it comes to the interpretation of our data. This question has been raised by other reviewers, and we went over it in greater detail in previous responses (Rev 1, Com 3; Rev 2, Com 1). In short, our data from alternatively expressed Gα-labeled G_s_ heterotrimers show no significant effect of changes in the expression level of Gα and γ subunits on the observed mobility (S6A Fig). We believe that expression level differences are not the main driver of the observed dynamic variability, but we now explicitly state it as a potential caveat in our manuscript. (l. 246-266)

3. The statement that “the wide distribution and lack of bias indicates that Gnat likely incorporates a number of different Gα subunits with different mobility patterns” would benefit from additional experimental support. For example, it would be informative to analyze Gβγ mobility in cells lacking endogenous Gα subunits, compared to conditions with endogenous or overexpressed Gα. Re-expression of individual Gα proteins in such a background could help determine whether Gα12 and Gα13 are indeed intrinsically slower, or whether mobility differences are influenced by relative abundance. No less exciting would be mobility assessment of Gnat in Ga12/13 KO cells.
**Response:** We thank the reviewer for the thoughtful suggestions. We decided to slightly temper the implication in our manuscript. While we were unable to facilitate such an expansion of the current study, the proposed experiments would be a great way to decipher the intricacies of G protein mobility in the future. (l. 185)

4. The final sentence of the abstract currently states: “These findings identify subtype-specific lateral membrane mobility of G proteins as a factor affecting their signaling dynamics in living cells.” As signaling dynamics are not directly measured in this study, this conclusion appears overstated. The sentence should be rephrased to reflect the descriptive nature of the data unless functional consequences are experimentally demonstrated.

**Response:** Thank you for the comment; the abstract has been adjusted accordingly. (l. 14-15)

5. How does overexpression of Gα and Gβ subunits affect the expression levels of labeled Gγ? Are there differences between G protein families in this regard? This point is important, as altered stoichiometry could influence the observed mobility.

**Response:** While the overexpression of Gβ_1_ could serve to stabilize the population of labeled Gγ_2,_ we do not believe there to be a causal link between the possible differences in Gβγ subunit interactions and the mobility data presented in our study, as the expression level of labeled Gγ_2_ was one of the main aspects of cell selection and remained consistent throughout the dataset.

6. Discussion: The statement “Their dynamic behavior is a key regulator of signal transfer dynamics” requires appropriate citation.

**Response:** Thank you for the comment; the abstract has been adjusted accordingly. (l. 191)

7. The reduced mobility of Gα12 and Gα13 could potentially be linked to stronger interactions with the actin cytoskeleton. This hypothesis could be tested experimentally, for example by disrupting the cytoskeleton and assessing whether mobility increases. Such an experiment would help connect the descriptive observations to a possible mechanistic explanation.
**Response:** Thank you for the comment. Cytoskeleton is indeed one of the potential interacting partners of G_12/13,_ and we have decided to include it in the manuscript. The investigation of this potential relationship would be a great component for future functional studies. (l. 204)

8. Before discussing mechanistic explanations for mobility differences, it is essential to establish whether these differences arise from variations in protein expression levels. If so, this would itself be an important finding that needs to be incorporated into the interpretation.

**Response:** The data presented in Supporting Figure 6A suggest that changes in the expression levels of Gα/γ do not play a major role in determining the mobility of G_s_ heterotrimers. At the same time, we are aware of the possible caveat, and we made sure to include it in the manuscript. (l. 246-266)

9. The authors speculate on potential reasons for the distinct behavior of Gα12 and Gα13. It may also be worth considering structural features such as their relatively long N-termini. These regions could influence interactions with Gβγ or membrane lipids (e.g., negatively charged phospholipids). If this is the case, altering membrane lipid composition might modulate mobility and could be explored experimentally.

**Response:** Our examination of the N-terminal structural elements possibly involved in mobility regulation has mostly focused on the distinct lipidation patterns and positive patch motifs of the different Gα subunits. Length is yet another aspect of N-terminal structure that could play a role in modulating mobility, and we decided to include it in the Discussion section of the manuscript. (l.224-230)

Overall, the study presents interesting observations; however, the current data are largely descriptive. Incorporating additional controls and/or tempering the conclusions would substantially strengthen the manuscript and improve the interpretation of these potentially important findings.

# Reviewer #4:

Significance:
The findings presented in this study by Alexey Bondar and colleagues provide important insight into an underappreciated layer of GPCR signaling regulation by demonstrating that heterotrimeric G proteins exhibit subtype-specific membrane mobility in living cells. While G protein signaling has traditionally been interpreted primarily through receptor coupling efficiency and downstream effector activation, these results support the emerging concept that the spatial and dynamic organization of signaling components within the plasma membrane itself can strongly influence signaling behavior. The markedly reduced mobility observed for Gα12- and Gα13-containing heterotrimers suggests that individual G protein subtypes may occupy distinct membrane environments or engage differently with membrane nanodomains, cytoskeletal structures, receptors, or signaling scaffolds. Such differences could directly affect receptor encounter rates, signaling kinetics, spatial confinement of signaling events, and access to downstream effectors. Importantly, these observations align with growing evidence that G proteins are highly dynamic signaling entities capable of membrane redistribution, compartmentalized signaling, and subtype-dependent trafficking behavior. Collectively, this work expands the current understanding of G Protein signaling beyond classical conformational activation models and highlights membrane mobility as a potentially important determinant of signaling specificity and cellular response dynamics. However, the manuscript lacks some significant information and contains contradictory facts compared with the already available data. The study is impactful and can be considered for publication in PLOS One after addressing the following concerns.
Major Concerns
1) Prior work shows that G proteins are not simply uniformly distributed cytosolic/membrane switches; they undergo subtype- and subunit-dependent membrane association, receptor-dependent immobilization, and activation-dependent redistribution. For example, Gβγ translocation kinetics vary markedly by γ-subtype, and βγ translocation to internal membranes can regulate downstream signaling, including Golgi-localized MAPK pathways. The authors have failed to gather relevant prior information published regarding G protein mobility in the manuscript. G protein mobility is a little old concept, and N. Gautam is one of the pioneers who worked on this. The authors would definitely benefit from reading those manuscripts.

**Response:** We thank the reviewer for their comment. We are aware of Professor Gautam’s contribution to the field, as well as other authors reporting on Gγ subunit involvement in G protein mobility. The focus of our study is the effect of the Gα subunit on lateral membrane mobility of G proteins, which has not been shown to necessarily correspond with intracellular translocation dynamics. In our attempt to stay focused on the topic at hand, we might have left out or oversimplified the previously reported data on G protein mobility. We have now made sure to adjust the “G protein localization” and “G protein mobility” sections of the manuscript to better reflect the previously reported findings and the broader narrative of G protein mobility and membrane localization in cellular signaling. (l. 32-52, 56-63, 92-96)

2) This is the most important interpretive limitation. The authors infer that the Gα subunit defines heterotrimer mobility, yet the measured particle is Gγ2-Halo. This inference is acceptable only if the authors demonstrate that most labeled Gγ2-Halo is incorporated into heterotrimers with the intended Gα rather than as free Gβγ, endogenous Gα complexes, or misassembled complexes. The “Gnat” control is useful but insufficient. They should include biochemical or imaging validation: co-expression dependence, membrane recruitment/retention, co-IP, BRET/FRET, or, at a minimum, functional receptor activation controls for representative Gα families. Additionally, multiple prior manuscripts have examined the mobility of different Gγ subtypes.

**Response:** Thank you for the comment. As we have stated in earlier responses, the functional heterotrimers of Gα_s_ [1], Gα_i1_ [2, 3, 4, 5], and Gα_o_ [2, 6] coupled with Gβ_1_γ_2_ have been shown in past publications. We have included additional data regarding heterotrimer mobility under adjusted conditions in Supplementary Figure 6A-B. This includes mobility data from G_s_ heterotrimers labeled at the Gα_s_ subunit, which exhibit mobility comparable to that of Gγ_2_-labeled G_s_, as well as data from G_s_ coexpressed with the V2R receptor that shows a decrease in mobility upon receptor stimulation consistent with previously shown activation of a functional G_s_ heterotrimer [1]. Together, this data suggests that our expression setup leads to functional assembly of the target heterotrimers. We were not able to create HaloTag versions of other Gα subunits because direct labeling of Gα subunits can affect their functional activity [5, 2], and newly created constructs require extensive biochemical characterization. Therefore, direct testing of other labeled Gα subunits remains beyond the scope of this study. (l. 252-257)

3) Non-tagged Gα and Gβ1 are CMV-driven, whereas Gγ2-Halo is minP-driven. This design aids sparse imaging but may produce nonphysiological Gα/Gβ/Gγ ratios. G proteins are highly stoichiometry-sensitive, and excess Gα or Gβγ can alter membrane occupancy, nanodomain partitioning, and trafficking. The manuscript needs quantitative expression controls, ideally showing comparable Gα/Gβ levels across conditions or at least normalizing membrane density beyond Gγ2-Halo density. If they are expressing heterotrimers, they need to use a polycistronic plasmid so the ratios match.

**Response:** Thank you for the comment. This question has been raised by other reviewers, and we went over it in greater detail in previous responses (Rev 1, Com 3; Rev 2, Com 1). To address the possible effect of expression level variability, we have included additional mobility data in Supporting Figure 6A. The data show two vastly different expression setups, one with HaloTag-labeled Gγ_2_ underexpressed under minP and Gα_s_ overexpressed under CMV, the other with HaloTag-labeled Gα_s_ underexpressed under minP and Gγ_2_ overexpressed under CMV promoter. Despite the stark difference in the ratio and expression level of the subunits, the heterotrimers exhibit comparable mobility. Because of that, we believe that expression levels are not a primary source of the observed dynamic variability in our study. At the same time, we made sure to include this as a possible limitation in our manuscript. (l. 246-266)

4) Gγ identity is a major determinant of Gβγ membrane affinity and translocation kinetics. Prior studies show that the Gγ subtype can strongly regulate βγ dissociation and translocation behavior after GPCR activation. Therefore, the conclusion should be narrowed: “Gα subtype alters the mobility of Gβ1γ2-containing heterotrimers,” not “heterotrimeric G proteins” broadly. Testing at least one additional Gγ subtype, even in a limited comparison such as Gγ7 or Gγ9 with Gαs versus Gα13, would substantially strengthen the paper. Rather than comparing a single Gγ type, it would be essential to examine mobility across Gγ subtypes, including fast, moderate, and slow. The authors should definitely think about changing the title

**Response:** We thank the reviewer for their comment. The primary focus of our study is the role of the Gα subunit subtype on G protein heterotrimer mobility, which has not been mapped as well as the role of the Gγ subunit. A wider-spanning study assessing a large number of different combinations of not only Gα and γ, but also β subunits will be a major future step in unveiling the broader narrative surrounding G protein mobility. Based on the reviewer’s suggestion, we have revised the title of the manuscript.

5) The introduction discusses membrane domains, but the discussion should better integrate the literature showing that Gα and Gβγ can shuttle between the plasma membrane, Golgi, ER, and endosomes, and that βγ translocation can be a signaling mechanism rather than only a localization artifact. This is directly relevant because reduced apparent TIRF mobility could reflect altered residence in PM nanodomains, receptor/effector complexes, or cytoskeletal corrals rather than intrinsic “slower heterotrimer diffusion.” Authors should remember that, not only Gα but also Gγ subtypes have lipid anchors that keep them on membranes. Gγ subtypes preferentially harbor two lipid anchor types, which facilitate membrane residence.

**Response:** Thank you for the comment. As mentioned in the response to your first comment, we have expanded the manuscript to better reflect the past research regarding intracellular trafficking and Gγ involvement in G protein mobility. (l. 60-63, 92-96)

6) The data indicate that several tracking parameters were adjusted “case-by-case,” including the quality threshold, linking distance, and gap-closing distance. This is a potential source of bias because slower and faster particles can be differentially affected by linking criteria. The authors should provide either fixed final parameters per condition, sensitivity analyses, or a reanalysis using identical parameters across all groups.

**Response:** We thank the reviewer for the thoughtful comment. The case-by-case adjustment of analysis parameters is indeed a potential point of bias introduction, but also an adaptation to the inherent sample variability in the dataset. The signal-to-noise ratio in our data can be affected by a plethora of elements, and adjusting the quality threshold is key to maintaining consistent particle recognition outcomes. Linking distance and gap-closing distance adjustment is a response to variability in G protein mobility. The equilibrium between oversegmentation and undersegmentation is key for proper analysis, but due to the cell-to-cell variability in our data, where the difference in mobility between two cells of the same condition can be up to threefold, this equilibrium is not always consistent. To assess whether our analysis approach played a major role in impacting the mobility outcomes of individual G proteins, we re-analyzed a representative set of data (the G_s_ and G_12_ data) using identical input values for both the quality thresholding (QT = 4) and linking criteria (LD = 0.7; GCD = 0.8) regardless of sample variability. The results of this re-analysis are included in Supporting Figure 7 and show that the statistically significant difference in mobility between the representative G_s_ and G_12_ groups remains despite the alternative analysis approach. (l. 343-347)

7) The figures appear to pool molecule tracks, but tracks from the same cell are not independent biological replicates. The authors mention extracting median values and reporting population sizes for each cell, yet Figure 3A appears to plot cell-level medians, whereas Figure 3B pools tracks. The authors should clearly state the unit of analysis: molecule track, cell, imaging day, or independent transfection. Statistical testing should be performed on biological/cell-level summary metrics, not on pooled tracks.

**Response:** Thank you for the comment. Figure 3B does indeed present pooled tracks as a depiction of value distribution. This approach does not allow us to perform robust statistical analysis, but we believe that it is still valuable as a showcase of otherwise hidden variability, such as specific mobility range bias or occurrence of distinct data subpopulations. We made sure to clarify all the statistical analysis approaches in the manuscript. (l. 146-148, 390-392, 518-526)

8) The claim that restricted Gα12/13 movement “likely points to physiological interactions with other signaling cascade members and regulators” is plausible but not proven. Alternative explanations include altered membrane lipid affinity, palmitoylation state, incomplete heterotrimer assembly, expression-level differences, cytoskeletal confinement, Gγ2-dependent behavior, and even endogenous Gγ expression levels. Authors should address these inconsistent and unsupported claims.

**Response:** Thank you for the comment. The purpose of this sentence was to point out that the bi-modal variability in our data could be a result of two separate underlying factors. We mentioned interactions with other signaling cascade members as one of the most probable explanations of increased low-mobility fractions, since this behavior has been observed in several other G proteins. At the same time, this claim cannot be fully supported by the current evidence, so we decided to remove it from the manuscript. (l. 161-165)

Minor issues
1) The phrase “unification and amplification of perceived extracellular chemical and physical stimuli” in the abstract is awkward. I would revise to: “Heterotrimeric G proteins transduce and amplify extracellular chemical and physical cues downstream of GPCRs.”

**Response:** Thank you, we revised the abstract to improve the flow of the text. (l. 2-6)

2) “antiobiotic/antimycotic” should be corrected to “antibiotic/antimycotic.”

**Response:** Thank you, we updated the manuscript accordingly. (l. 282)

3) The manuscripts would again benefit from overall grammar, spelling, and wording revision. For example, the conclusion should avoid “point out to” and use “point to.”

**Response:** We thank the reviewer for their comment; we made sure to revise the manuscript to improve clarity.

# **References**

1. Bondar A, Jang W, Sviridova E, Lambert NA. Components of the Gs signaling cascade exhibit distinct changes in mobility and membrane domain localization upon β2-adrenergic receptor activation. Traffic. 2020;21: 324–332. doi:10.1111/tra.12724
2. Bondar A, Lazar J. Dissociated GαGTP and Gβγ Protein Subunits Are the Major Activated Form of Heterotrimeric Gi/o Proteins. Journal of Biological Chemistry. 2014;289: 1271–1281. doi:10.1074/jbc.M113.493643
3. Sungkaworn T, Jobin M-L, Burnecki K, Weron A, Lohse MJ, Calebiro D. Single-molecule imaging reveals receptor–G protein interactions at cell surface hot spots. Nature. 2017;550: 543–547. doi:10.1038/nature24264
4. Bünemann M, Frank M, Lohse MJ. Gi protein activation in intact cells involves subunit rearrangement rather than dissociation. Proceedings of the National Academy of Sciences. 2003;100: 16077–16082. doi:10.1073/pnas.2536719100
5. Gibson SK, Gilman AG. Giα and Gβ subunits both define selectivity of G protein activation by α2-adrenergic receptors. Proceedings of the National Academy of Sciences. 2006;103: 212–217. doi:10.1073/pnas.0509763102
6. Frank M, Thümer L, Lohse MJ, Bünemann M. G Protein Activation without Subunit Dissociation Depends on a Gαi-specific Region*. Journal of Biological Chemistry. 2005;280: 24584–24590. doi:10.1074/jbc.M414630200
