# Supplementary material for: Heterotrimeric G proteins containing Gβ1γ2 subunits exhibit subtype-specific mobility differences in live cells
Source: PLoS One. 2026 Sep 29;21(9):e0359304. doi: 10.1371/journal.pone.0359304 (PMC13623102; doi:10.1371/journal.pone.0359304)
Supplement: Supplementary file 12 [file pone.0359304.s017.docx]

**Reviewer #1**

1. The current limitations regarding the use of Gγ2-HaloTag as a reporter should be stated more explicitly. In particular, the authors cannot directly determine what fraction of labeled Gβ1γ2 is associated with the exogenously expressed Gα subtype, as opposed to endogenous Gα proteins or free Gβγ. I therefore recommend adding a clear statement to the Limitations section, for example: “We cannot directly determine the fraction of labeled Gβ1γ2 associated with the exogenously expressed Gα subtype versus endogenous Gα or free Gβγ.”

**Response:** We thank the reviewer for the suggestion. We have decided to directly state and address this possible caveat in the Limitations section of the manuscript. At the same time, we believe that the supplemental data from the G_s_ protein suggest that the mobilities reported by Gγ_2_-HaloTag are relevant indicators for individual G proteins (S6A-B Fig). Additionally, if we assume an issue in the assembly of G_12_ or G_13_, resulting in either endogenous Gα pairing or free Gβγ, the reported mobilities should shift towards the values observed in G_nat_. Instead, we see that the values observed in G_12_ and G_13_ sharply diverge from those observed in G_nat_ (Fig 3A), indicating that the mobility difference between G_12/13_ and G_s_, at the very least, cannot be explained by misassembly alone. (l.243-253)

2. For the experiments using Gα13-IRES-mCherry and Gαq-IRES-mCherry, the Methods should clearly state whether the mCherry signal was used as a criterion for cell selection. If it was used, the selection criteria should be described. If it was not used, this should also be clarified, particularly because the expression constructs for Gα13 and Gαq differ from those used for the other Gα subunits.

**Response:** Thank you for the suggestion. We have included a statement addressing the (non)usage of mCherry signal in the cell selection process in the manuscript. (l. 314-315)

3. In the Methods, the membrane density used for cell selection is described as approximately “0.1–0.3 mol/µm2.” This appears to be a unit error and should presumably be “0.1–0.3 molecules/µm2.” Please confirm and correct the unit accordingly.

**Response:** Thank you. This was a typographic error from earlier versions of the manuscript that went unnoticed. It has now been corrected. (l. 313)

**Reviewer #4**

The authors adequately validate that Gγ2-Halo reports the intended heterotrimer, given the scope. The Gαs-HaloTag comparison, the V2R/AVP activation control, and the Gαs-ΔCt2 data collectively support functional assembly. One suggestion to strengthen the argument at no experimental cost: the authors have a strong internal control they do not use. If Gγ2-Halo were failing to incorporate with exogenous Gα12/Gα13, those conditions should converge on the Gnat value (~0.32 µm²/s); instead, they diverge sharply (0.14 and 0.20 µm²/s). This logic directly argues against the misassembly scenario and belongs in the Limitations section.

**Response:** We highly appreciate the thoughtful suggestion. We decided to clearly articulate this point in the updated Limitations section. (l. 243-253)

It is worth noting in the text that the fixed linking distance (0.7 µm) sits at the low end of the range used, which, if anything, biases against the faster groups.

**Response:** Thank you for the comment. We included a statement regarding analysis parameters in the manuscript. (l. 338-340)

- However, the abstract overstates the comparison involving Gq. It states that Gα12/Gα13-containing heterotrimers show markedly reduced mobility "compared to those containing Gαi/o, Gαs, and Gαq." This is not what the statistics show: in Fig 3A, Gq is assigned BC and shares group C with G12 and G13, and in Fig 4B, Gq groups with G12 and G13 (B). The Results text is appropriately careful ("somewhat in between these groups"), but the abstract is not. Please either remove Gαq from that sentence or qualify it (e.g., "with Gαq intermediate").

**Response:** The comparison between G_q_ and the other subunits is indeed complicated, differing depending on the metric (fraction of diffusion states/overall diffusion coefficient/diffusion state-specific diffusion coefficient). The interpretation of the results in the abstract was too narrow and has been corrected. (l. 10)

- Residual overgeneralization in headings and the conclusion. The title and abstract were narrowed to Gβ1γ2-containing heterotrimers, but the Results heading ("Different families of G proteins exhibit distinct mobility patterns in live cells"), the closing sentence of the Discussion ("strong differences in mobility of different types of heterotrimeric G proteins"), and the phrase "in the broader context of G protein mobility" still generalize beyond the tested pairing. Please align these with the revised title.

**Response:** Thank you. The latter parts of the manuscript have been updated to reflect the adjusted characterization. (l. 85, 109, 168-169, 223)

- The single Gγ subtype is not listed as a limitation. Given that Gγ identity itself determines Gβγ membrane behavior, the restriction to Gβ1γ2 warrants a fourth item in the Limitations section, alongside expression level, density, and cell type.

**Response:** Thank you for the suggestion. We have decided to expand the Limitations chapter to better articulate this constraint in result interpretation. (l. 264-267)

- Statistical reporting is incomplete. The Methods define ε² and rank-biserial correlation, but no test statistics, p-values, or effect sizes appear in the Results or in a supporting table (only the regression output is reported). Please report these, state which test was applied to each figure (Kruskal-Wallis/Dunn vs Mann-Whitney), and specify the α and correction used for the compact letter display in Figs 3A and 4B.

**Response:** Thank you for the suggestion. The details of all statistical analyses are included in the supplementary table accompanying the publication, as mandated by PLOS ONE guidelines, which may not be available during the review process. At the same time, we agree that important statistical indicators should be included directly in the manuscript. To preserve readability, we have decided to include all the important statistical indicators in their corresponding figure legends. (l. 130-131, 155-156, 519-520, 531-532, 535-538, 540-545, 547-549, 552-555)

- S6 and S7 carry a heavy interpretive burden for the descriptive data. S6A is cited in response to four separate reviewer concerns about expression levels, yet the legend states that no statistical inference was performed due to low sample size. This is understandable, but the wording throughout ("comparable mobility," "no significant effect") should be softened, since the absence of testing is not evidence of equivalence. Reporting n and the observed ranges for each S6 condition would help.

**Response:** We thank the reviewer for the suggestion. Statistical analysis was not possible in S6A and S6B Fig due to the low effect size and the low sample size, respectively. The pairwise comparison in S6C Fig includes proper statistical analysis. We made sure to better explain this in the manuscript, as well as include statistical indicators, n, and observed ranges where applicable. (l. 535-538, 540-545, 547-549)

- Clarify the basis for the diffusion-state percentages. The values quoted in the Results (59/60/57% vs 44/41/41%) should be identified as pooled-track fractions or as means/medians of per-cell fractions, consistent with the new statement that cell-level medians are the statistical unit.

**Response:** Thank you. During the assessment of this part of the manuscript, we have noticed an oversight on our part, where in-text values of the mean per-cell fractions were not updated according to the analysis approach adjusted during the second round of review. We have made sure to correct this, as well as implement your suggestion in the manuscript. (l. 138-140, 162)

- Membrane density is reported as 0.14-0.20 molecules/µm² in the Results but as ~0.1-0.3 mol/um² in the TIRF Methods; please reconcile and use µm² consistently.

**Response:** The numerical discrepancy arises because the Results show the calculated average membrane density values of the different subunits, also shown in S1 Table, whereas the TIRF Methods report on the approximate acceptable range of values used for cell selection, within which almost all analyzed cells fall. The use of mol/um² in this instance was a typographical error and has been corrected. (l. 313)

- "lipidation motive" → "lipidation motif" (Discussion).

**Response:** Thank you. We adjusted the manuscript accordingly. (l. 190)

- Inconsistent naming of the truncation mutant: "Gαs-ΔCt2" vs. "Gs-ΔCt2" (Methods, Limitations, S6 legend).

**Response:** Thank you for the comment. We made sure to adjust the manuscript to correctly refer to the truncated mutant of the Gα_s_ subunit as "Gα_s_-ΔCt2", and the resulting heterotrimer containing Gα_s_-ΔCt2 as "G_s_-ΔCt2", consistent with other heterotrimer references in the manuscript. (l.238-239, 539)

I think the manuscript is ready for publication after these edits.
