# Supplementary material for: Heterotrimeric G proteins containing Gβ1γ2 subunits exhibit subtype-specific mobility differences in live cells
Source: PLoS One. 2026 Sep 29;21(9):e0359304. doi: 10.1371/journal.pone.0359304 (PMC13623102; doi:10.1371/journal.pone.0359304)
Supplement: Peer review history

# Peer review: Decision Letter 0

**DOI:** 10.1371/journal.pone.0359304.r001

**Authors:** Yosuke Senju

18 May 2026

Dear Dr. Bondar,

Thank you for submitting your manuscript to PLOS ONE. After careful consideration, we feel that it has merit but does not fully meet PLOS ONE’s publication criteria as it currently stands. Therefore, we invite you to submit a revised version of the manuscript that addresses the points raised during the review process.

Please submit your revised manuscript by Jul 02 2026 11:59PM. If you will need more time than this to complete your revisions, please reply to this message or contact the journal office at plosone@plos.org. When you're ready to submit your revision, log on to https://www.editorialmanager.com/pone/ and select the 'Submissions Needing Revision' folder to locate your manuscript file.

- A letter that responds to each point raised by the academic editor and reviewer(s). You should upload this letter as a separate file labeled 'Response to Reviewers'.
- A marked-up copy of your manuscript that highlights changes made to the original version. You should upload this as a separate file labeled 'Revised Manuscript with Track Changes'.
- An unmarked version of your revised paper without tracked changes. You should upload this as a separate file labeled 'Manuscript'.

If you would like to make changes to your financial disclosure, please include your updated statement in your cover letter. Guidelines for resubmitting your figure files are available below the reviewer comments at the end of this letter.

If applicable, we recommend that you deposit your laboratory protocols in protocols.io to enhance the reproducibility of your results. Protocols.io assigns your protocol its own identifier (DOI) so that it can be cited independently in the future. For instructions see: https://journals.plos.org/plosone/s/submission-guidelines#loc-laboratory-protocols. Additionally, PLOS ONE offers an option for publishing peer-reviewed Lab Protocol articles, which describe protocols hosted on protocols.io. Read more information on sharing protocols at https://plos.org/protocols?utm_medium=editorial-email&utm_source=authorletters&utm_campaign=protocols.

As the corresponding author, your ORCID iD is verified in the submission system and will appear in the published article. PLOS supports the use of ORCID, and we encourage all coauthors to register for an ORCID iD and use it as well. Please encourage your coauthors to verify their ORCID iD within the submission system before final acceptance, as unverified ORCID iDs will not appear in the published article. *Only*  the individual author can complete the verification step; PLOS staff *cannot*  verify ORCID iDs on behalf of authors.

We look forward to receiving your revised manuscript.

Kind regards,

Yosuke Senju, Ph.D.

Academic Editor

PLOS One

Journal Requirements:

When submitting your revision, we need you to address these additional requirements.

1. Please ensure that your manuscript meets PLOS ONE's style requirements, including those for file naming. The PLOS ONE style templates can be found at https://journals.plos.org/plosone/s/file?id=wjVg/PLOSOne_formatting_sample_main_body.pdf and https://journals.plos.org/plosone/s/file?id=ba62/PLOSOne_formatting_sample_title_authors_affiliations.pdf

2. Please note that PLOS One has specific guidelines on code sharing for submissions in which author-generated code underpins the findings in the manuscript. In these cases, we expect all author-generated code to be made available without restrictions upon publication of the work. Please review our guidelines at https://journals.plos.org/plosone/s/materials-and-software-sharing#loc-sharing-code and ensure that your code is shared in a way that follows best practice and facilitates reproducibility and reuse.

3. Thank you for stating in your Funding Statement:

[This work was supported by the Czech Science Foundation grant 26-22607K (T.F.).].

Please provide an amended statement that declares *all* the funding or sources of support (whether external or internal to your organization) received during this study, as detailed online in our guide for authors at http://journals.plos.org/plosone/s/submit-now.  Please also include the statement “There was no additional external funding received for this study.” in your updated Funding Statement.

Please include your amended Funding Statement within your cover letter. We will change the online submission form on your behalf.

4. Please include captions for all of your Supporting Information files at the end of your manuscript, and update any in-text citations to match accordingly. Please see our Supporting Information guidelines for more information: http://journals.plos.org/plosone/s/supporting-information.

If the reviewer comments include a recommendation to cite specific previously published works, please review and evaluate these publications to determine whether they are relevant and should be cited. There is no requirement to cite these works unless the editor has indicated otherwise.

[Note: HTML markup is below. Please do not edit.]

Reviewers' comments:

Reviewer's Responses to Questions

**Comments to the Author**

1. Is the manuscript technically sound, and do the data support the conclusions?

Reviewer #1: Partly

Reviewer #2: No

Reviewer #3: No

Reviewer #4: Partly

**********

2. Has the statistical analysis been performed appropriately and rigorously?

Reviewer #1: No

Reviewer #2: Yes

Reviewer #3: No

Reviewer #4: No

**********

3. Have the authors made all data underlying the findings in their manuscript fully available?

The PLOS Data policy

Reviewer #1: Yes

Reviewer #2: Yes

Reviewer #3: Yes

Reviewer #4: Yes

**********

4. Is the manuscript presented in an intelligible fashion and written in standard English?

Reviewer #1: Yes

Reviewer #2: Yes

Reviewer #3: Yes

Reviewer #4: Yes

**********

Reviewer #1: This manuscript investigates whether the lateral mobility of heterotrimeric G proteins on the plasma membrane differs depending on the type of Gα subunit, using live-cell TIRF single-molecule imaging and single-particle tracking. The authors introduced untagged Gα subunits, Gβ1, and low-expression Gγ2-HaloTag into CHO-K1 cells, and used Gγ2-HaloTag as a common reporter to compare the membrane diffusion of heterotrimeric G proteins presumed to contain each Gα subtype. They report that G proteins containing Gα12 and Gα13 show lower diffusion coefficients than those containing Gαs, Gαi1, and Gαo. They further report that Gα12- and Gα13-containing G proteins have a higher fraction of restricted diffusion components classified as immobile and confined diffusion.

This study may provide useful insight into the spatiotemporal regulation of GPCR–G protein signaling. In particular, the observation that Gα12/Gα13-containing heterotrimers exhibit low mobility is interesting. However, several important points require additional data or clarification to sufficiently support the authors’ claims.

Major comments

1. The relationship to previous studies and the novelty of the present work should be clarified

The central concept of this study is that the membrane mobility of G proteins is heterogeneous and can be altered by subunit composition, membrane domains, and interactions with GPCRs, which has already been demonstrated in previous single-molecule studies. For example, heterogeneous mobility of G proteins, membrane microdomain localization of Gq heterotrimers, changes in membrane localization and diffusion behavior depending on combinations of Gα and Gγ subunits, and reduced G-protein mobility upon interaction with GPCRs have all been reported previously (Oerez JMB 2006; Abankwa J Cell Sci 2007; van Hemert J Cell Sci 2010; Mystek Cells 2019...).

Therefore, the authors should describe the claim that “the type of Gα subunit determines the mobility of heterotrimeric G proteins” not as an entirely new concept, but rather as an extension of previous studies. The specific novelty of the present study appears to lie in the side-by-side comparison of multiple Gα subtypes under the same experimental conditions, particularly in showing that heterotrimers containing Gα12 and Gα13 exhibit low mobility. This point should be clearly stated in the Introduction and Discussion.

2. It is necessary to validate that Gγ2-HaloTag reflects the mobility of the intended Gα-containing heterotrimers

In this study, the authors use Gγ2-HaloTag as a common reporter and estimate the mobility of heterotrimeric G proteins containing each Gα subtype by co-expressing the respective Gα subunits. However, it has not been sufficiently demonstrated that Gγ2-HaloTag preferentially forms heterotrimers with the intended exogenous Gα subunits.

Gγ2-HaloTag may associate with endogenous Gα subunits or may exist as free Gβγ. Therefore, it is unclear whether the observed differences in mobility reflect differences among heterotrimers containing each Gα subtype, or instead reflect differences in association efficiency with exogenous Gα, competition with endogenous Gα, or the proportion of free Gβγ.

The authors should demonstrate, by co-immunoprecipitation, membrane localization analysis, expression-level comparison, functional complementation, or another independent approach, that Gγ2-HaloTag is an appropriate reporter for heterotrimers containing the intended Gα subunits. At minimum, the authors should provide representative imaging examples for cells expressing each Gα subunit and clearly discuss this point as a limitation of the study.

3. The effects of expression levels and membrane molecular density should be evaluated more rigorously

The authors state that Gγ2-HaloTag was used under low-expression conditions, but the explanation is insufficient regarding how the labeled molecule density, Gα subunit expression level, and cell-to-cell variability in each condition may affect the diffusion coefficients. Differences in expression level or membrane density may influence molecular crowding on the membrane, the stoichiometry of Gβγ and Gα, competition with endogenous G proteins, and the fraction of free Gβγ.

The membrane density of Gγ2-HaloTag in each condition should be clearly shown in the main text or in a major supplementary figure, and the authors should demonstrate that there is no correlation between membrane density and diffusion coefficient. It is also necessary to show that the expression levels of the different Gα subunits are comparable, or at least within a range that does not substantially affect the interpretation of the results. In particular, because the Gα13-IRES-mCherry and Gαq-IRES-mCherry constructs differ from the other pcDNA3.1 expression constructs, the authors should explain the expression levels and criteria used for cell selection.

4. The statistical unit and sample size should be clarified

In the present manuscript, it is not sufficiently clear whether the statistical analyses were performed based on individual tracks or on summary statistics such as median values per cell.

The authors should clearly state what each point in each figure represents and whether the unit of statistical testing was the track, the cell, or the independent experiment. Ideally, statistical analyses should be performed using cell-level values, and the numbers of independent transfection experiments, analyzed cells, and tracks should be clearly indicated for each condition.

Minor comments

1. Since Gα itself was not directly labeled in this study, expressions such as “Gα12 mobility” should be avoided. The measured target should be described more precisely, for example as “the mobility of Gα12-containing heterotrimers inferred from Gγ2-HaloTag tracking.”

2. In Fig. 4, the authors should indicate whether statistical comparisons were performed for the proportions of restricted and non-restricted movement.

3. In the Methods, the authors state that fixed parameters were used, whereas they also state that the detection threshold, linking distance, and gap-closing distance in the TrackMate analysis were adjusted for each sample. The authors should clarify which parameters were fixed and which were optimized for each condition.

4. The term “antiobiotic/antimycotic” in the Methods appears to be a typographical error for “antibiotic/antimycotic” and should be corrected.

Reviewer #2: In the very broad and important field of cellular signalling, G protein cascades have exceptional significance not only because they are involved in nearly all physiological processes in humans, but also because they are among the most widely targeted pathways in therapeutic interventions. Therefore, the manuscript by Kuchynka and collaborators may potentially arouse interest among a wide audience. While the authors present a bunch of interesting and well elaborated data, several important methodological limitations significantly weaken the support of the conclusions drawn. As a result, the whole story is too preliminary to be publishable in its current form, and the authors should consider a few issues listed below.

- First of all, the levels of overexpressed non-tagged G proteins within target cells are to large extent unknown. In fact, the authors provide membrane densities in Table S1, but this is most probably based on the signal from G gamma subunit. Unfortunately, the methodology is not presented, and the table description is misleading since the specified G proteins were not directly labelled. Apparently, the real stoichiometry of all the G protein subunits within cells was not taken into consideration, although it may severely influence behaviour of these proteins (i.e. oligomerization, diffusion, membrane affinities, etc.).

- As the authors aptly point out in the discussion, it should be expected that the diffusion of G proteins strongly depends on the level of their lipidation. It is already known that overexpression of proteins in mammalian cells may overload the intracellular enzymatic system responsible for lipidation/delipidation and thus result in partial lipidation (or artefactual change in lipidation pattern) of the proteins of interest (see e.g. 10.3390/membranes11090715). Thus, without experimental confirmation (e.g. via “acyl-RAC” method, see DOI: 10.1194/jlr.D011106) that the overexpressed G proteins exhibit posttranslational modifications patterns identical to physiologically relevant counterparts the data presented within the manuscript would have very limited significance.

- To further clarify the importance of individual structural elements of G proteins (as discussed within l. 177-184 and l.194-206), site-directed mutagenesis could be performed in order to generate proteins that lack particular functionalities.

- Would the behaviour of G proteins vary depending on host cell type? The presented data were generated using only one cell line and thus raise a question whether the observed effects are cell-specific.

- Minor issues include: the colour code for G protein heterotrimer models on Figs 1 and 2 should be defined; caveolae have biochemical features of lipid rafts and thus should be considered as specialized subset of lipid rafts (see e. g. DOI: 10.1038/nrc3915 and refer it to l.41-48 of the main text); the panel B of Figure 4 is redundant – using two distinguishable patterns (on top of the already used colour code) on the panel A would do a better job; referring to more recent papers in the field of G protein raft partitioning is highly advisable (e.g. DOI: 10.3390/biomedicines11020557, DOI: 10.1007/978-3-540-72843-6, to name a few).

Reviewer #3: The authors report G protein subfamily-specific differences in lateral membrane mobility, with Gα12- and Gα13-containing heterotrimers displaying particularly slow diffusion. The manuscript is concise, clearly written, and supported by well-designed figures. However, the current study remains rather preliminary and would benefit from additional experimental support and/or a more cautious interpretation of the data.

1. Results (lines 84–86): The authors state that direct labeling of Gα subunits can affect their interaction with Gβγ, and therefore use labeled Gγ2 as a universal mobility readout. It would be important to clarify whether labeling of Gγ might itself influence Gα–Gβγ interactions. Even if Gγ is uniformly labeled, it remains possible that such labeling differentially affects distinct Gα subunits. In addition, does Gα mobility change depending on whether Gγ is labeled or not? These points should be addressed experimentally or, at minimum, discussed as important caveats.

2. Figure 3A: To what extent could the slower diffusion of Gα12- and Gα13-containing complexes be related to differences in expression levels or abundance? How comparable are the expression levels of the various Gα proteins, and how does altered expression of individual subunits affect mobility? These are critical aspects that should be clarified before drawing conclusions about intrinsic subtype-specific mobility.

3. The statement that “the wide distribution and lack of bias indicates that Gnat likely incorporates a number of different Gα subunits with different mobility patterns” would benefit from additional experimental support. For example, it would be informative to analyze Gβγ mobility in cells lacking endogenous Gα subunits, compared to conditions with endogenous or overexpressed Gα. Re-expression of individual Gα proteins in such a background could help determine whether Gα12 and Gα13 are indeed intrinsically slower, or whether mobility differences are influenced by relative abundance. No less exciting would be mobility assessment of Gnat in Ga12/13 KO cells.

4. The final sentence of the abstract currently states: “These findings identify subtype-specific lateral membrane mobility of G proteins as a factor affecting their signaling dynamics in living cells.” As signaling dynamics are not directly measured in this study, this conclusion appears overstated. The sentence should be rephrased to reflect the descriptive nature of the data unless functional consequences are experimentally demonstrated.

5. How does overexpression of Gα and Gβ subunits affect the expression levels of labeled Gγ? Are there differences between G protein families in this regard? This point is important, as altered stoichiometry could influence the observed mobility.

6. Discussion: The statement “Their dynamic behavior is a key regulator of signal transfer dynamics” requires appropriate citation.

7. The reduced mobility of Gα12 and Gα13 could potentially be linked to stronger interactions with the actin cytoskeleton. This hypothesis could be tested experimentally, for example by disrupting the cytoskeleton and assessing whether mobility increases. Such an experiment would help connect the descriptive observations to a possible mechanistic explanation.

8. Before discussing mechanistic explanations for mobility differences, it is essential to establish whether these differences arise from variations in protein expression levels. If so, this would itself be an important finding that needs to be incorporated into the interpretation.

9. The authors speculate on potential reasons for the distinct behavior of Gα12 and Gα13. It may also be worth considering structural features such as their relatively long N-termini. These regions could influence interactions with Gβγ or membrane lipids (e.g., negatively charged phospholipids). If this is the case, altering membrane lipid composition might modulate mobility and could be explored experimentally.

Overall, the study presents interesting observations; however, the current data are largely descriptive. Incorporating additional controls and/or tempering the conclusions would substantially strengthen the manuscript and improve the interpretation of these potentially important findings.

Reviewer #4: Significance:

The findings presented in this study by Alexey Bondar and colleagues provide important insight into an underappreciated layer of GPCR signaling regulation by demonstrating that heterotrimeric G proteins exhibit subtype-specific membrane mobility in living cells. While G protein signaling has traditionally been interpreted primarily through receptor coupling efficiency and downstream effector activation, these results support the emerging concept that the spatial and dynamic organization of signaling components within the plasma membrane itself can strongly influence signaling behavior. The markedly reduced mobility observed for Gα12- and Gα13-containing heterotrimers suggests that individual G protein subtypes may occupy distinct membrane environments or engage differently with membrane nanodomains, cytoskeletal structures, receptors, or signaling scaffolds. Such differences could directly affect receptor encounter rates, signaling kinetics, spatial confinement of signaling events, and access to downstream effectors. Importantly, these observations align with growing evidence that G proteins are highly dynamic signaling entities capable of membrane redistribution, compartmentalized signaling, and subtype-dependent trafficking behavior. Collectively, this work expands the current understanding of G Protein signaling beyond classical conformational activation models and highlights membrane mobility as a potentially important determinant of signaling specificity and cellular response dynamics. However, the manuscript lacks some significant information and contains contradictory facts compared with the already available data. The study is impactful and can be considered for publication in PLOS One after addressing the following concerns.

Major Concerns

1) Prior work shows that G proteins are not simply uniformly distributed cytosolic/membrane switches; they undergo subtype- and subunit-dependent membrane association, receptor-dependent immobilization, and activation-dependent redistribution. For example, Gβγ translocation kinetics vary markedly by γ-subtype, and βγ translocation to internal membranes can regulate downstream signaling, including Golgi-localized MAPK pathways. The authors have failed to gather relevant prior information published regarding G protein mobility in the manuscript. G protein mobility is a little old concept, and N. Gautam is one of the pioneers who worked on this. The authors would definitely benefit from reading those manuscripts.

2) This is the most important interpretive limitation. The authors infer that the Gα subunit defines heterotrimer mobility, yet the measured particle is Gγ2-Halo. This inference is acceptable only if the authors demonstrate that most labeled Gγ2-Halo is incorporated into heterotrimers with the intended Gα rather than as free Gβγ, endogenous Gα complexes, or misassembled complexes. The “Gnat” control is useful but insufficient. They should include biochemical or imaging validation: co-expression dependence, membrane recruitment/retention, co-IP, BRET/FRET, or, at a minimum, functional receptor activation controls for representative Gα families. Additionally, multiple prior manuscripts have examined the mobility of different Gγ subtypes.

3) Non-tagged Gα and Gβ1 are CMV-driven, whereas Gγ2-Halo is minP-driven. This design aids sparse imaging but may produce nonphysiological Gα/Gβ/Gγ ratios. G proteins are highly stoichiometry-sensitive, and excess Gα or Gβγ can alter membrane occupancy, nanodomain partitioning, and trafficking. The manuscript needs quantitative expression controls, ideally showing comparable Gα/Gβ levels across conditions or at least normalizing membrane density beyond Gγ2-Halo density. If they are expressing heterotrimers, they need to use a polycistronic plasmid so the ratios match.

4) Gγ identity is a major determinant of Gβγ membrane affinity and translocation kinetics. Prior studies show that the Gγ subtype can strongly regulate βγ dissociation and translocation behavior after GPCR activation. Therefore, the conclusion should be narrowed: “Gα subtype alters the mobility of Gβ1γ2-containing heterotrimers,” not “heterotrimeric G proteins” broadly. Testing at least one additional Gγ subtype, even in a limited comparison such as Gγ7 or Gγ9 with Gαs versus Gα13, would substantially strengthen the paper. Rather than comparing a single Gγ type, it would be essential to examine mobility across Gγ subtypes, including fast, moderate, and slow. The authors should definitely think about changing the title

5) The introduction discusses membrane domains, but the discussion should better integrate the literature showing that Gα and Gβγ can shuttle between the plasma membrane, Golgi, ER, and endosomes, and that βγ translocation can be a signaling mechanism rather than only a localization artifact. This is directly relevant because reduced apparent TIRF mobility could reflect altered residence in PM nanodomains, receptor/effector complexes, or cytoskeletal corrals rather than intrinsic “slower heterotrimer diffusion.” Authors should remember that, not only Gα but also Gγ subtypes have lipid anchors that keep them on membranes. Gγ subtypes preferentially harbor two lipid anchor types, which facilitate membrane residence.

6) The data indicate that several tracking parameters were adjusted “case-by-case,” including the quality threshold, linking distance, and gap-closing distance. This is a potential source of bias because slower and faster particles can be differentially affected by linking criteria. The authors should provide either fixed final parameters per condition, sensitivity analyses, or a reanalysis using identical parameters across all groups.

7) The figures appear to pool molecule tracks, but tracks from the same cell are not independent biological replicates. The authors mention extracting median values and reporting population sizes for each cell, yet Figure 3A appears to plot cell-level medians, whereas Figure 3B pools tracks. The authors should clearly state the unit of analysis: molecule track, cell, imaging day, or independent transfection. Statistical testing should be performed on biological/cell-level summary metrics, not on pooled tracks.

8) The claim that restricted Gα12/13 movement “likely points to physiological interactions with other signaling cascade members and regulators” is plausible but not proven. Alternative explanations include altered membrane lipid affinity, palmitoylation state, incomplete heterotrimer assembly, expression-level differences, cytoskeletal confinement, Gγ2-dependent behavior, and even endogenous Gγ expression levels. Authors should address these inconsistent and unsupported claims.

Minor issues

1) The phrase “unification and amplification of perceived extracellular chemical and physical stimuli” in the abstract is awkward. I would revise to: “Heterotrimeric G proteins transduce and amplify extracellular chemical and physical cues downstream of GPCRs.”

2) “antiobiotic/antimycotic” should be corrected to “antibiotic/antimycotic.”

3) The manuscripts would again benefit from overall grammar, spelling, and wording revision. For example, the conclusion should avoid “point out to” and use “point to.”

**********

what does this mean?). If published, this will include your full peer review and any attached files.

If you choose “no”, your identity will remain anonymous but your review may still be made public.

**Do you want your identity to be public for this peer review?**  For information about this choice, including consent withdrawal, please see our Privacy Policy

Reviewer #1: No

Reviewer #2: No

Reviewer #3: No

Reviewer #4: No

**********

[NOTE: If reviewer comments were submitted as an attachment file, they will be attached to this email and accessible via the submission site. Please log into your account, locate the manuscript record, and check for the action link "View Attachments". If this link does not appear, there are no attachment files.]

To ensure your figures meet our technical requirements, please review our figure guidelines: https://journals.plos.org/plosone/s/figures

You may also use PLOS’s free figure tool, NAAS, to help you prepare publication quality figures: https://journals.plos.org/plosone/s/figures#loc-tools-for-figure-preparation.

NAAS will assess whether your figures meet our technical requirements by comparing each figure against our figure specifications.

Attachment Submitted filename: Reviewer Report - PONE-D-26-18579.docx

# Author comment: Author response to Decision Letter 1

**DOI:** 10.1371/journal.pone.0359304.r002

6 Aug 2026

Reviewer #1

: This manuscript investigates whether the lateral mobility of heterotrimeric G proteins on the plasma membrane differs depending on the type of Gα subunit, using live-cell TIRF single-molecule imaging and single-particle tracking. The authors introduced untagged Gα subunits, Gβ1, and low-expression Gγ2-HaloTag into CHO-K1 cells, and used Gγ2-HaloTag as a common reporter to compare the membrane diffusion of heterotrimeric G proteins presumed to contain each Gα subtype. They report that G proteins containing Gα12 and Gα13 show lower diffusion coefficients than those containing Gαs, Gαi1, and Gαo. They further report that Gα12- and Gα13-containing G proteins have a higher fraction of restricted diffusion components classified as immobile and confined diffusion.

This study may provide useful insight into the spatiotemporal regulation of GPCR–G protein signaling. In particular, the observation that Gα12/Gα13-containing heterotrimers exhibit low mobility is interesting. However, several important points require additional data or clarification to sufficiently support the authors’ claims.

Major comments

1. The relationship to previous studies and the novelty of the present work should be clarified

The central concept of this study is that the membrane mobility of G proteins is heterogeneous and can be altered by subunit composition, membrane domains, and interactions with GPCRs, which has already been demonstrated in previous single-molecule studies. For example, heterogeneous mobility of G proteins, membrane microdomain localization of Gq heterotrimers, changes in membrane localization and diffusion behavior depending on combinations of Gα and Gγ subunits, and reduced G-protein mobility upon interaction with GPCRs have all been reported previously (Oerez JMB 2006; Abankwa J Cell Sci 2007; van Hemert J Cell Sci 2010; Mystek Cells 2019...).

Therefore, the authors should describe the claim that “the type of Gα subunit determines the mobility of heterotrimeric G proteins” not as an entirely new concept, but rather as an extension of previous studies. The specific novelty of the present study appears to lie in the side-by-side comparison of multiple Gα subtypes under the same experimental conditions, particularly in showing that heterotrimers containing Gα12 and Gα13 exhibit low mobility. This point should be clearly stated in the Introduction and Discussion.

Response: Thank you for the comment; we modified the manuscript to better reflect the relationship of our results to previously published data. (l. 92-96)

2. It is necessary to validate that Gγ2-HaloTag reflects the mobility of the intended Gα-containing heterotrimers

In this study, the authors use Gγ2-HaloTag as a common reporter and estimate the mobility of heterotrimeric G proteins containing each Gα subtype by co-expressing the respective Gα subunits. However, it has not been sufficiently demonstrated that Gγ2-HaloTag preferentially forms heterotrimers with the intended exogenous Gα subunits.

Gγ2-HaloTag may associate with endogenous Gα subunits or may exist as free Gβγ. Therefore, it is unclear whether the observed differences in mobility reflect differences among heterotrimers containing each Gα subtype, or instead reflect differences in association efficiency with exogenous Gα, competition with endogenous Gα, or the proportion of free Gβγ.

The authors should demonstrate, by co-immunoprecipitation, membrane localization analysis, expression-level comparison, functional complementation, or another independent approach, that Gγ2-HaloTag is an appropriate reporter for heterotrimers containing the intended Gα subunits. At minimum, the authors should provide representative imaging examples for cells expressing each Gα subunit and clearly discuss this point as a limitation of the study.

Response: We thank the reviewer for their recommendation of relevant controls. We, as well as others, have previously shown that Gαs [1], Gαi1 [2, 3, 4, 5], and Gαo [2, 6] subunits form functional heterotrimers with Gβ1γ2 dimers and can be activated by GPCRs. Though we could not carry out all of the proposed experiments, we included additional supporting information in Supporting Figures 5 and 6. These include, as suggested, images of cells under each condition, as well as a mobility comparison between Gs protein labeled at the Gγ subunit and directly at the Gαs subunit. The data show that both labeling approaches produce the same heterotrimer mobility outcomes. Additionally, the data in Supporting Figure 6B showcase the decrease in the mobility of Gs containing labeled Gγ2-HaloTag in the presence of an activated V2R receptor, consistent with previously shown activation of a functional Gs heterotrimer [1]. This suggests that overexpressed Gαs forms functional heterotrimers with Gβ1 and Gγ2-HaloTag, indicating that the observed high values of the diffusion coefficient are not caused by reduced binding of Gγ2-HaloTag with certain overexpressed Gα subunits, such as Gαs. We were not able to create HaloTag versions of other Gα subunits because direct labeling of Gα subunits can affect their functional activity [5, 2], and newly created constructs require extensive biochemical characterization. Therefore, direct testing of other labeled Gα subunits remains beyond the scope of this study. We have also added a Limitations section to the manuscript. (l. 246-271)

3. The effects of expression levels and membrane molecular density should be evaluated more rigorously

The authors state that Gγ2-HaloTag was used under low-expression conditions, but the explanation is insufficient regarding how the labeled molecule density, Gα subunit expression level, and cell-to-cell variability in each condition may affect the diffusion coefficients. Differences in expression level or membrane density may influence molecular crowding on the membrane, the stoichiometry of Gβγ and Gα, competition with endogenous G proteins, and the fraction of free Gβγ.

The membrane density of Gγ2-HaloTag in each condition should be clearly shown in the main text or in a major supplementary figure, and the authors should demonstrate that there is no correlation between membrane density and diffusion coefficient. It is also necessary to show that the expression levels of the different Gα subunits are comparable, or at least within a range that does not substantially affect the interpretation of the results. In particular, because the Gα13-IRES-mCherry and Gαq-IRES-mCherry constructs differ from the other pcDNA3.1 expression constructs, the authors should explain the expression levels and criteria used for cell selection.

Response: The manuscript was expanded by a linear regression analysis of membrane density and diffusion coefficient values (S5I Fig), showing no correlation between the two. Though, as correctly pointed out by the reviewer, this approach is limited as it only incorporates the membrane density of Gγ2-HaloTag, and not the overexpressed Gα and β. This weakness is, in part, offset by the data showcased in Supporting Figure 6A, showing that Gs heterotrimers containing a HaloTag-labeled Gαs subunit expressed under the minP promoter exhibit similar mobility to those with an overexpressed unlabeled Gα subunit. This suggests that the difference in expression levels of both Gα and Gγ does not have a critical impact on the observed mobility. At the same time, if we assume expression level differences as the main driver of the decreased mobility in G12 and G13 through effects such as membrane crowding, that would necessitate strongly and consistently increased expression levels of Gα12 and Gα13 compared to the other subunits, which we are unaware of there being a precedent for. At the same time, we are aware of the limitations of this approach, and we now explicitly acknowledge it in the manuscript. (l.246-266)

Cell selection was based primarily on single-molecule analysis suitability, specifically, the lack of non-specific fluorescence and Gγ2-HaloTag membrane density, as well as healthy cell appearance. We made sure to include this information in the manuscript as well. (l. 318-319)

4. The statistical unit and sample size should be clarified

In the present manuscript, it is not sufficiently clear whether the statistical analyses were performed based on individual tracks or on summary statistics such as median values per cell.

The authors should clearly state what each point in each figure represents and whether the unit of statistical testing was the track, the cell, or the independent experiment. Ideally, statistical analyses should be performed using cell-level values, and the numbers of independent transfection experiments, analyzed cells, and tracks should be clearly indicated for each condition.

Response: Thank you; the number of experiments, analyzed cells, and tracks has been included in Supporting Table 2, and the manuscript has been adjusted accordingly. (l. 141-148, 390-392, 518-526). We agree that only individual cells can serve as biological replicates and would like to emphasize that all statistical analyses were performed using cell-level values.

Minor comments

1. Since Gα itself was not directly labeled in this study, expressions such as “Gα12 mobility” should be avoided. The measured target should be described more precisely, for example as “the mobility of Gα12-containing heterotrimers inferred from Gγ2-HaloTag tracking.”

Response: We thank the reviewer for the suggestion; we revised our manuscript accordingly. (l. 144, 208, 247-248)

2. In Fig. 4, the authors should indicate whether statistical comparisons were performed for the proportions of restricted and non-restricted movement.

Response: We have updated Fig 4 to better showcase our results and allow for robust statistical analysis. (l. 167-175)

3. In the Methods, the authors state that fixed parameters were used, whereas they also state that the detection threshold, linking distance, and gap-closing distance in the TrackMate analysis were adjusted for each sample. The authors should clarify which parameters were fixed and which were optimized for each condition.

Response: We thank the reviewer for the insightful comments; we revised the manuscript to improve clarity and better explain our approach. (l. 115, 336-337)

4. The term “antiobiotic/antimycotic” in the Methods appears to be a typographical error for “antibiotic/antimycotic” and should be corrected.

Response: Thank you, we updated the manuscript accordingly. (l. 282)

Reviewer #2:

In the very broad and important field of cellular signalling, G protein cascades have exceptional significance not only because they are involved in nearly all physiological processes in humans, but also because they are among the most widely targeted pathways in therapeutic interventions. Therefore, the manuscript by Kuchynka and collaborators may potentially arouse interest among a wide audience. While the authors present a bunch of interesting and well elaborated data, several important methodological limitations significantly weaken the support of the conclusions drawn. As a result, the whole story is too preliminary to be publishable in its current form, and the authors should consider a few issues listed below.

- First of all, the levels of overexpressed non-tagged G proteins within target cells are to large extent unknown. In fact, the authors provide membrane densities in Table S1, but this is most probably based on the signal from G gamma subunit. Unfortunately, the methodology is not presented, and the table description is misleading since the specified G proteins were not directly labelled. Apparently, the real stoichiometry of all the G protein subunits within cells was not taken into consideration, although it may severely influence behaviour of these proteins (i.e. oligomerization, diffusion, membrane affinities, etc.).

Response: We highly appreciate the comment. Indeed, expression levels of the proteins of interest are important for the interpretation of the results. We have now performed additional experiments (S6 Fig) and show that direct labeling of the Gαs subunit yields diffusion coefficients comparable to those determined through Gγ2 labeling. Since this combination of Gαs with Gβ1γ2 dimers yields higher diffusion coefficient values than observed for the Gα12 subunit, we believe that our results can’t be explained solely by expression level differences. Additionally, we have now provided representative images of cells with all construct combinations and evaluated the dependence of Gγ2 density and diffusion coefficient. We have found that Gγ2 density does not directly correlate with diffusion coefficient values. At the same time, we are aware of the limitations of our approach and have now explicitly indicated this, as well as our methodology behind membrane density measurements in the text of the manuscript. (l. 246-266, 559-561)

- As the authors aptly point out in the discussion, it should be expected that the diffusion of G proteins strongly depends on the level of their lipidation. It is already known that overexpression of proteins in mammalian cells may overload the intracellular enzymatic system responsible for lipidation/delipidation and thus result in partial lipidation (or artefactual change in lipidation pattern) of the proteins of interest (see e.g. 10.3390/membranes11090715). Thus, without experimental confirmation (e.g. via “acyl-RAC” method, see DOI: 10.1194/jlr.D011106) that the overexpressed G proteins exhibit posttranslational modifications patterns identical to physiologically relevant counterparts the data presented within the manuscript would have very limited significance.

Response: Thank you for the comment. After reviewing the presented literature, we have decided to include partial lipidation as a possible element of variability in the Discussion section of the manuscript. At the same time, we believe that lipidation alone cannot explain the variability observed in our results for a few reasons. Partial lipidation would likely result in subpopulations of G proteins with distinct mobility patterns, especially so in Gα12, which only contains a single palmitoylation site, meaning it can only exist within palmitoylated and non-palmitoylated states. Broadly speaking, partial lipidation of subunits should lead to a decrease in membrane affinity and an increase in lateral membrane mobility. This would point to our results showing not decreased mobility in G12, but increased mobility in all the other subunits. This is possible, but the differences in lipidation would likely produce vastly different outcomes in myristoylated subunits Gαi1 and Gαo, in which myristoylation is mandatory for proper trafficking and membrane attachment, compared to subunits that are only palmitoylated, such as Gαs and Gαq. Another reason why we believe that lipidation variance related to overexpression is not a major driver of the observed mobility differences is that Gs heterotrimers exhibit comparable mobility in the presence of both overexpressed and underexpressed Gαs (S6A Fig). (l. 221-223)

- To further clarify the importance of individual structural elements of G proteins (as discussed within l. 177-184 and l.194-206), site-directed mutagenesis could be performed in order to generate proteins that lack particular functionalities.

Response: Thank you for the thoughtful suggestion. While unable to carry out the proposed steps, in Supporting Figure 6A, we have included data from heterotrimers containing a Gαs subunit mutant lacking two C-terminal amino acids. Our data suggest its mobility might be slightly elevated compared to regular Gs heterotrimers, which could correlate with reduced efficiency in protein-protein interactions, particularly GPCR binding, due to the truncated C-terminus. Other mutant variants of various subunits would be an interesting direction for future research. (l. 257-260)

- Would the behaviour of G proteins vary depending on host cell type? The presented data were generated using only one cell line and thus raise a question whether the observed effects are cell-specific.

Response: We have added mobility measurements of the Gs protein from the HEK-293 cell line in Supporting Figure 6C. These data suggest a significant difference in the mobility between the two cell lines and point to an increased importance of cell line identity on the overall mobility of membrane proteins. We additionally discuss this as a possible point of limitation in the manuscript and argue that absolute values of diffusion coefficients from experimental systems intrinsically have limited value, but the differences between them point to important trends in mobility of G protein heterotrimers. (l. 267-271)

- Minor issues include: the colour code for G protein heterotrimer models on Figs 1 and 2 should be defined; caveolae have biochemical features of lipid rafts and thus should be considered as specialized subset of lipid rafts (see e. g. DOI: 10.1038/nrc3915 and refer it to l.41-48 of the main text); the panel B of Figure 4 is redundant – using two distinguishable patterns (on top of the already used colour code) on the panel A would do a better job; referring to more recent papers in the field of G protein raft partitioning is highly advisable (e.g. DOI: 10.3390/biomedicines11020557, DOI: 10.1007/978-3-540-72843-6, to name a few).

Response: We thank the reviewer for the insightful comments; we revised the manuscript to improve clarity and figure presentation. (l. 70,119-120; 46-58, 65; 169-175; 32-58)

Reviewer #3

: The authors report G protein subfamily-specific differences in lateral membrane mobility, with Gα12- and Gα13-containing heterotrimers displaying particularly slow diffusion. The manuscript is concise, clearly written, and supported by well-designed figures. However, the current study remains rather preliminary and would benefit from additional experimental support and/or a more cautious interpretation of the data.

1. Results (lines 84–86): The authors state that direct labeling of Gα subunits can affect their interaction with Gβγ, and therefore use labeled Gγ2 as a universal mobility readout. It would be important to clarify whether labeling of Gγ might itself influence Gα–Gβγ interactions. Even if Gγ is uniformly labeled, it remains possible that such labeling differentially affects distinct Gα subunits. In addition, does Gα mobility change depending on whether Gγ is labeled or not? These points should be addressed experimentally or, at minimum, discussed as important caveats.

Response: Thank you for the comment. Fluorescent labeling can indeed cause various issues with protein interaction, folding, or steric hindrance, meaning reporter design inextricably comes with a certain degree of invasiveness. Our labeling approach has been focused on minimizing this degree of invasiveness by inserting the fluorescent protein at the N-terminus of the Gγ subunit, which is the furthest-facing part of the heterotrimer from both the membrane and the receptor interaction centers, such as the Gα C-terminus. At the same time, using a single labeling approach across experiments avoids the introduction of additional variability that comes with using multiple different insertion sites. Despite that, this approach still carries a risk of functional interference. Because of that, we have included a mobility comparison between Gs heterotrimers labeled at the Gγ subunit and the Gα subunit (S6A Fig). The data suggest that the mobility of Gαs-labeled heterotrimers does not significantly differ from the Gs mobility observed in our study. (l. 252-254)

2. Figure 3A: To what extent could the slower diffusion of Gα12- and Gα13-containing complexes be related to differences in expression levels or abundance? How comparable are the expression levels of the various Gα proteins, and how does altered expression of individual subunits affect mobility? These are critical aspects that should be clarified before drawing conclusions about intrinsic subtype-specific mobility.

Response: We thank the reviewer for the comment. Expression level differences are an important variable when it comes to the interpretation of our data. This question has been raised by other reviewers, and we went over it in greater detail in previous responses (Rev 1, Com 3; Rev 2, Com 1). In short, our data from alternatively expressed Gα-labeled Gs heterotrimers show no significant effect of changes in the expression level of Gα and γ subunits on the observed mobility (S6A Fig). We believe that expression level differences are not the main driver of the observed dynamic variability, but we now explicitly state it as a potential caveat in our manuscript. (l. 246-266)

3. The statement that “the wide distribution and lack of bias indicates that Gnat likely incorporates a number of different Gα subunits with different mobility patterns” would benefit from additional experimental support. For example, it would be informative to analyze Gβγ mobility in cells lacking endogenous Gα subunits, compared to conditions with endogenous or overexpressed Gα. Re-expression of individual Gα proteins in such a background could help determine whether Gα12 and Gα13 are indeed intrinsically slower, or whether mobility differences are influenced by relative abundance. No less exciting would be mobility assessment of Gnat in Ga12/13 KO cells.

Response: We thank the reviewer for the thoughtful suggestions. We decided to slightly temper the implication in our manuscript. While we were unable to facilitate such an expansion of the current study, the proposed experiments would be a great way to decipher the intricacies of G protein mobility in the future. (l. 185)

4. The final sentence of the abstract currently states: “These findings identify subtype-specific lateral membrane mobility of G proteins as a factor affecting their signaling dynamics in living cells.” As signaling dynamics are not directly measured in this study, this conclusion appears overstated. The sentence should be rephrased to reflect the descriptive nature of the data unless functional consequences are experimentally demonstrated.

Response: Thank you for the comment; the abstract has been adjusted accordingly. (l. 14-15)

5. How does overexpression of Gα and Gβ subunits affect the expression levels of labeled Gγ? Are there differences between G protein families in this regard? This point is important, as altered stoichiometry could influence the observed mobility.

Response: While the overexpression of Gβ1 could serve to stabilize the population of labeled Gγ2, we do not believe there to be a causal link between the possible differences in Gβγ subunit interactions and the mobility data presented in our study, as the expression level of labeled Gγ2 was one of the main aspects of cell selection and remained consistent throughout the dataset.

6. Discussion: The statement “Their dynamic behavior is a key regulator of signal transfer dynamics” requires appropriate citation.

Response: Thank you for the comment; the abstract has been adjusted accordingly. (l. 191)

7. The reduced mobility of Gα12 and Gα13 could potentially be linked to stronger interactions with the actin cytoskeleton. This hypothesis could be tested experimentally, for example by disrupting the cytoskeleton and assessing whether mobility increases. Such an experiment would help connect the descriptive observations to a possible mechanistic explanation.

Response: Thank you for the comment. Cytoskeleton is indeed one of the potential interacting partners of G12/13, and we have decided to include it in the manuscript. The investigation of this potential relationship would be a great component for future functional studies. (l. 204)

8. Before discussing mechanistic explanations for mobility differences, it is essential to establish whether these differences arise from variations in protein expression levels. If so, this would itself be an important finding that needs to be incorporated into the interpretation.

Response: The data presented in Supporting Figure 6A suggest that changes in the expression levels of Gα/γ do not play a major role in determining the mobility of Gs heterotrimers. At the same time, we are aware of the possible caveat, and we made sure to include it in the manuscript. (l. 246-266)

9. The authors speculate on potential reasons for the distinct behavior of Gα12 and Gα13. It may also be worth considering structural features such as their relatively long N-termini. These regions could influence interactions with Gβγ or membrane lipids (e.g., negatively charged phospholipids). If this is the case, altering membrane lipid composition might modulate mobility and could be explored experimentally.

Response: Our examination of the N-terminal structural elements possibly involved in mobility regulation has mostly focused on the distinct lipidation patterns and positive patch motifs of the different Gα subunits. Length is yet another aspect of N-terminal structure that could play a role in modulating mobility, and we decided to include it in the Discussion section of the manuscript. (l.224-230)

Overall, the study presents interesting observations; however, the current data are largely descriptive. Incorporating additional controls and/or tempering the conclusions would substantially strengthen the manuscript and improve the interpretation of these potentially important findings.

Reviewer #4:

Significance:

The findings presented in this study by Alexey Bondar and colleagues provide important insight into an underappreciated layer of GPCR signaling regulation by demonstrating that heterotrimeric G proteins exhibit subtype-specific membrane mobility in living cells. While G protein signaling has traditionally been interpreted primarily through receptor coupling efficiency and downstream effector activation, these results support the emerging concept that the spatial and dynamic organization of signaling components within the plasma membrane itself can strongly influence signaling behavior. The markedly reduced mobility observed for Gα12- and Gα13-containing heterotrimers suggests that individual G protein subtypes may occupy distinct membrane environments or engage differently with membrane nanodomains, cytoskeletal structures, receptors, or signaling scaffolds. Such differences could directly affect receptor encounter rates, signaling kinetics, spatial confinement of signaling events, and access to downstream effectors. Importantly, these observations align with growing evidence that G proteins are highly dynamic signaling entities capable of membrane redistribution, compartmentalized signaling, and subtype-dependent trafficking behavior. Collectively, this work expands the current understanding of G Protein signaling beyond classical conformational activation models and highlights membrane mobility as a potentially important determinant of signaling specificity and cellular response dynamics. However, the manuscript lacks some significant information and contains contradictory facts compared with the already available data. The study is impactful and can be considered for publication in PLOS One after addressing the following concerns.

Major Concerns

1) Prior work shows that G proteins are not simply uniformly distributed cytosolic/membrane switches; they undergo subtype- and subunit-dependent membrane association, receptor-dependent immobilization, and activation-dependent redistribution. For example, Gβγ translocation kinetics vary markedly by γ-subtype, and βγ translocation to internal membranes can regulate downstream signaling, including Golgi-localized MAPK pathways. The authors have failed to gather relevant prior information published regarding G protein mobility in the manuscript. G protein mobility is a little old concept, and N. Gautam is one of the pioneers who worked on this. The authors would definitely benefit from reading those manuscripts.

Response: We thank the reviewer for their comment. We are aware of Professor Gautam’s contribution to the field, as well as other authors reporting on Gγ subunit involvement in G protein mobility. The focus of our study is the effect of the Gα subunit on lateral membrane mobility of G proteins, which has not been shown to necessarily correspond with intracellular translocation dynamics. In our attempt to stay focused on the topic at hand, we might have left out or oversimplified the previously reported data on G protein mobility. We have now made sure to adjust the “G protein localization” and “G protein mobility” sections of the manuscript to better reflect the previously reported findings and the broader narrative of G protein mobility and membrane localization in cellular signaling. (l. 32-52, 56-63, 92-96)

2) This is the most important interpretive limitation. The authors infer that the Gα subunit defines heterotrimer mobility, yet the measured particle is Gγ2-Halo. This inference is acceptable only if the authors demonstrate that most labeled Gγ2-Halo is incorporated into heterotrimers with the intended Gα rather than as free Gβγ, endogenous Gα complexes, or misassembled complexes. The “Gnat” control is useful but insufficient. They should include biochemical or imaging validation: co-expression dependence, membrane recruitment/retention, co-IP, BRET/FRET, or, at a minimum, functional receptor activation controls for representative Gα families. Additionally, multiple prior manuscripts have examined the mobility of different Gγ subtypes.

Response: Thank you for the comment. As we have stated in earlier responses, the functional heterotrimers of Gαs [1], Gαi1 [2, 3, 4, 5], and Gαo [2, 6] coupled with Gβ1γ2 have been shown in past publications. We have included additional data regarding heterotrimer mobility under adjusted conditions in Supplementary Figure 6A-B. This includes mobility data from Gs heterotrimers labeled at the Gαs subunit, which exhibit mobility comparable to that of Gγ2-labeled Gs, as well as data from Gs coexpressed with the V2R receptor that shows a decrease in mobility upon receptor stimulation consistent with previously shown activation of a functional Gs heterotrimer [1]. Together, this data suggests that our expression setup leads to functional assembly of the target heterotrimers. We were not able to create HaloTag versions of other Gα subunits because direct labeling of Gα subunits can affect their functional activity [5, 2], and newly created constructs require extensive biochemical characterization. Therefore, direct testing of other labeled Gα subunits remains beyond the scope of this study. (l. 252-257)

3) Non-tagged Gα and Gβ1 are CMV-driven, whereas Gγ2-Halo is minP-driven. This design aids sparse imaging but may produce nonphysiological Gα/Gβ/Gγ ratios. G proteins are highly stoichiometry-sensitive, and excess Gα or Gβγ can alter membrane occupancy, nanodomain partitioning, and trafficking. The manuscript needs quantitative expression controls, ideally showing comparable Gα/Gβ levels across conditions or at least normalizing membrane density beyond Gγ2-Halo density. If they are expressing heterotrimers, they need to use a polycistronic plasmid so the ratios match.

Response: Thank you for the comment. This question has been raised by other reviewers, and we went over it in greater detail in previous responses (Rev 1, Com 3; Rev 2, Com 1). To address the possible effect of expression level variability, we have included additional mobility data in Supporting Figure 6A. The data show two vastly different expression setups, one with HaloTag-labeled Gγ2 underexpressed under minP and Gαs overexpressed under CMV, the other with HaloTag-labeled Gαs underexpressed under minP and Gγ2 overexpressed under CMV promoter. Despite the stark difference in the ratio and expression level of the subunits, the heterotrimers exhibit comparable mobility. Because of that, we believe that expression levels are not a primary source of the observed dynamic variability in our study. At the same time, we made sure to include this as a possible limitation in our manuscript. (l. 246-266)

4) Gγ identity is a major determinant of Gβγ membrane affinity and translocation kinetics. Prior studies show that the Gγ subtype can strongly regulate βγ dissociation and translocation behavior after GPCR activation. Therefore, the conclusion should be narrowed: “Gα subtype alters the mobility of Gβ1γ2-containing heterotrimers,” not “heterotrimeric G proteins” broadly. Testing at least one additional Gγ subtype, even in a limited comparison such as Gγ7 or Gγ9 with Gαs versus Gα13, would substantially strengthen the paper. Rather than comparing a single Gγ type, it would be essential to examine mobility across Gγ subtypes, including fast, moderate, and slow. The authors should definitely think about changing the title

Response: We thank the reviewer for their comment. The primary focus of our study is the role of the Gα subunit subtype on G protein heterotrimer mobility, which has not been mapped as well as the role of the Gγ subunit. A wider-spanning study assessing a large number of different combinations of not only Gα and γ, but also β subunits will be a major future step in unveiling the broader narrative surrounding G protein mobility. Based on the reviewer’s suggestion, we have revised the title of the manuscript.

5) The introduction discusses membrane domains, but the discussion should better integrate the literature showing that Gα and Gβγ can shuttle between the plasma membrane, Golgi, ER, and endosomes, and that βγ translocation can be a signaling mechanism rather than only a localization artifact. This is directly relevant because reduced apparent TIRF mobility could reflect altered residence in PM nanodomains, receptor/effector complexes, or cytoskeletal corrals rather than intrinsic “slower heterotrimer diffusion.” Authors should remember that, not only Gα but also Gγ subtypes have lipid anchors that keep them on membranes. Gγ subtypes preferentially harbor two lipid anchor types, which facilitate membrane residence.

Response: Thank you for the comment. As mentioned in the response to your first comment, we have expanded the manuscript to better reflect the past research regarding intracellular trafficking and Gγ involvement in G protein mobility. (l. 60-63, 92-96)

6) The data indicate that several tracking parameters were adjusted “case-by-case,” including the quality threshold, linking distance, and gap-closing distance. This is a potential source of bias because slower and faster particles can be differentially affected by linking criteria. The authors should provide either fixed final parameters per condition, sensitivity analyses, or a reanalysis using identical parameters across all groups.

Response: We thank the reviewer for the thoughtful comment. The case-by-case adjustment of analysis parameters is indeed a potential point of bias introduction, but also an adaptation to the inherent sample variability in the dataset. The signal-to-noise ratio in our data can be affected by a plethora of elements, and adjusting the quality threshold is key to maintaining consistent particle recognition outcomes. Linking distance and gap-closing distance adjustment is a response to variability in G protein mobility. The equilibrium between oversegmentation and undersegmentation is key for proper analysis, but due to the cell-to-cell variability in our data, where the difference in mobility between two cells of the same condition can be up to threefold, this equilibrium is not always consistent. To assess whether our analysis approach played a major role in impacting the mobility outcomes of individual G proteins, we re-analyzed a representative set of data (the Gs and G12 data) using identical input values for both the quality thresholding (QT = 4) and linking criteria (LD = 0.7; GCD = 0.8) regardless of sample variability. The results of this re-analysis are included in Supporting Figure 7 and show that the statistically significant difference in mobility between the representative Gs and G12 groups remains despite the alternative analysis approach. (l. 343-347)

7) The figures appear to pool molecule tracks, but tracks from the same cell are not independent biological replicates. The authors mention extracting median values and reporting population sizes for each cell, yet Figure 3A appears to plot cell-level medians, whereas Figure 3B pools tracks. The authors should clearly state the unit of analysis: molecule track, cell, imaging day, or independent transfection. Statistical testing should be performed on biological/cell-level summary metrics, not on pooled tracks.

Response: Thank you for the comment. Figure 3B does indeed present pooled tracks as a depiction of value distribution. This approach does not allow us to perform robust statistical analysis, but we believe that it is still valuable as a showcase of otherwise hidden variability, such as specific mobility range bias or occurrence of distinct data subpopulations. We made sure to clarify all the statistical analysis approaches in the manuscript. (l. 146-148, 390-392, 518-526)

8) The claim that restricted Gα12/13 movement “likely points to physiological interactions with other signaling cascade members and regulators” is plausible but not proven. Alternative explanations include altered membrane lipid affinity, palmitoylation state, incomplete heterotrimer assembly, expression-level differences, cytoskeletal confinement, Gγ2-dependent behavior, and even endogenous Gγ expression levels. Authors should address these inconsistent and unsupported claims.

Response: Thank you for the comment. The purpose of this sentence was to point out that the bi-modal variability in our data could be a result of two separate underlying factors. We mentioned interactions with other signaling cascade members as one of the most probable explanations of increased low-mobility fractions, since this behavior has been observed in several other G proteins. At the same time, this claim cannot be fully supported by the current evidence, so we decided to remove it from the manuscript. (l. 161-165)

Minor issues

1) The phrase “unification and amplification of perceived extracellular chemical and physical stimuli” in the abstract is awkward. I would revise to: “Heterotrimeric G proteins transduce and amplify extracellular chemical and physical cues downstream of GPCRs.”

Response: Thank you, we revised the abstract to improve the flow of the text. (l. 2-6)

2) “antiobiotic/antimycotic” should be corrected to “antibiotic/antimycotic.”

Response: Thank you, we updated the manuscript accordingly. (l. 282)

3) The manuscripts would again benefit from overall grammar, spelling, and wording revision. For example, the conclusion should avoid “point out to” and use “point to.”

Response: We thank the reviewer for their comment; we made sure to revise the manuscript to improve clarity.

References

1. Bondar A, Jang W, Sviridova E, Lambert NA. Components of the Gs signaling cascade exhibit distinct changes in mobility and membrane domain localization upon β2-adrenergic receptor activation. Traffic. 2020;21: 324–332. doi:10.1111/tra.12724

2. Bondar A, Lazar J. Dissociated GαGTP and Gβγ Protein Subunits Are the Major Activated Form of Heterotrimeric Gi/o Proteins. Journal of Biological Chemistry. 2014;289: 1271–1281. doi:10.1074/jbc.M113.493643

3. Sungkaworn T, Jobin M-L, Burnecki K, Weron A, Lohse MJ, Calebiro D. Single-molecule imaging reveals receptor–G protein interactions at cell surface hot spots. Nature. 2017;550: 543–547. doi:10.1038/nature24264

4. Bünemann M, Frank M, Lohse MJ. Gi protein activation in intact cells involves subunit rearrangement rather than dissociation. Proceedings of the National Academy of Sciences. 2003;100: 16077–16082. doi:10.1073/pnas.2536719100

5. Gibson SK, Gilman AG. Giα and Gβ subunits both define selectivity of G protein activation by α2-adrenergic receptors. Proceedings of the National Academy of Sciences. 2006;103: 212–217. doi:10.1073/pnas.0509763102

6. Frank M, Thümer L, Lohse MJ, Bünemann M. G Protein Activation without Subunit Dissociation Depends on a Gαi-specific Region*. Journal of Biological Chemistry. 2005;280: 24584–24590. doi:10.1074/jbc.M414630200

Attachment Submitted filename: Response to reviewers.docx

# Peer review: Decision Letter 1

**DOI:** 10.1371/journal.pone.0359304.r003

**Authors:** Yosuke Senju

24 Aug 2026

Dear Dr. Bondar,

Thank you for submitting your manuscript to PLOS One. After careful consideration, we feel that it has merit but does not fully meet PLOS One’s publication criteria as it currently stands. Therefore, we invite you to submit a revised version of the manuscript that addresses the points raised during the review process.

Please submit your revised manuscript by Oct 08 2026 11:59PM. If you will need more time than this to complete your revisions, please reply to this message or contact the journal office at plosone@plos.org. When you're ready to submit your revision, log on to https://www.editorialmanager.com/pone/ and select the 'Submissions Needing Revision' folder to locate your manuscript file.

- A letter that responds to each point raised by the academic editor and reviewer(s). You should upload this letter as a separate file labeled 'Response to Reviewers'.
- A marked-up copy of your manuscript that highlights changes made to the original version. You should upload this as a separate file labeled 'Revised Manuscript with Track Changes'.
- An unmarked version of your revised paper without tracked changes. You should upload this as a separate file labeled 'Manuscript'.

If you would like to make changes to your financial disclosure, please include your updated statement in your cover letter. Guidelines for resubmitting your figure files are available below the reviewer comments at the end of this letter.

If applicable, we recommend that you deposit your laboratory protocols in protocols.io to enhance the reproducibility of your results. Protocols.io assigns your protocol its own identifier (DOI) so that it can be cited independently in the future. For instructions see: https://journals.plos.org/plosone/s/submission-guidelines#loc-laboratory-protocols. Additionally, PLOS One offers an option for publishing peer-reviewed Lab Protocol articles, which describe protocols hosted on protocols.io. Read more information on sharing protocols at https://plos.org/protocols?utm_medium=editorial-email&utm_source=authorletters&utm_campaign=protocols.

As the corresponding author, your ORCID iD is verified in the submission system and will appear in the published article. PLOS supports the use of ORCID, and we encourage all coauthors to register for an ORCID iD and use it as well. Please encourage your coauthors to verify their ORCID iD within the submission system before final acceptance, as unverified ORCID iDs will not appear in the published article. *Only*  the individual author can complete the verification step; PLOS staff *cannot*  verify ORCID iDs on behalf of authors.

We look forward to receiving your revised manuscript.

Kind regards,

Yosuke Senju, Ph.D.

Academic Editor

PLOS One

Journal Requirements:

If the reviewer comments include a recommendation to cite specific previously published works, please review and evaluate these publications to determine whether they are relevant and should be cited. There is no requirement to cite these works unless the editor has indicated otherwise.

Please review your reference list to ensure that it is complete and correct. If you have cited papers that have been retracted, please include the rationale for doing so in the manuscript text, or remove these references and replace them with relevant current references. Any changes to the reference list should be mentioned in the rebuttal letter that accompanies your revised manuscript. If you need to cite a retracted article, indicate the article’s retracted status in the References list and also include a citation and full reference for the retraction notice.

[Note: HTML markup is below. Please do not edit.]

Reviewers' comments:

Reviewer's Responses to Questions

**Comments to the Author**

Reviewer #1: (No Response)

Reviewer #2: All comments have been addressed

Reviewer #3: All comments have been addressed

Reviewer #4: All comments have been addressed

**********

2. Is the manuscript technically sound, and do the data support the conclusions?

Reviewer #1: Partly

Reviewer #2: Yes

Reviewer #3: Yes

Reviewer #4: Partly

**********

3. Has the statistical analysis been performed appropriately and rigorously?

Reviewer #1: Yes

Reviewer #2: Yes

Reviewer #3: (No Response)

Reviewer #4: Yes

**********

4. Have the authors made all data underlying the findings in their manuscript fully available?

The PLOS Data policy

Reviewer #1: Yes

Reviewer #2: Yes

Reviewer #3: (No Response)

Reviewer #4: Yes

**********

5. Is the manuscript presented in an intelligible fashion and written in standard English?

Reviewer #1: Yes

Reviewer #2: Yes

Reviewer #3: (No Response)

Reviewer #4: Yes

**********

Reviewer #1: The authors have adequately addressed most of the concerns raised in the previous round of review, and the manuscript has been substantially improved. However, a few issues remain and should be addressed before the manuscript can be considered for publication.

1. The current limitations regarding the use of Gγ2-HaloTag as a reporter should be stated more explicitly. In particular, the authors cannot directly determine what fraction of labeled Gβ1γ2 is associated with the exogenously expressed Gα subtype, as opposed to endogenous Gα proteins or free Gβγ. I therefore recommend adding a clear statement to the Limitations section, for example:

“We cannot directly determine the fraction of labeled Gβ1γ2 associated with the exogenously expressed Gα subtype versus endogenous Gα or free Gβγ.”

2. For the experiments using Gα13-IRES-mCherry and Gαq-IRES-mCherry, the Methods should clearly state whether the mCherry signal was used as a criterion for cell selection. If it was used, the selection criteria should be described. If it was not used, this should also be clarified, particularly because the expression constructs for Gα13 and Gαq differ from those used for the other Gα subunits.

3. In the Methods, the membrane density used for cell selection is described as approximately “0.1–0.3 mol/µm2.” This appears to be a unit error and should presumably be “0.1–0.3 molecules/µm2.” Please confirm and correct the unit accordingly.

Reviewer #2: I would like to thank the authors for addressing all the issues raised by the authors. The manuscript improved considerably and is now ready for publication in PLoS One.

Reviewer #3: The authors have adequately addressed my comments. I appreciate their thoughtful revisions and have no further concerns.

Reviewer #4: The authors have responded constructively and thoroughly to the previous round of review. The literature framing is much improved, the statistical unit is now better, the unsupported mechanistic claim has been removed, and the new Limitations section is a welcome addition. The new supporting data (S5-S7) address the most important concerns within the scope of this study. I have only a few residual points, most of them textual.

The expanded "G protein localization" and "G protein mobility" sections now situate the work appropriately, and the added references (Gautam and colleagues, Mystek, Abankwa) better reflect the field.

The authors adequately validate that Gγ2-Halo reports the intended heterotrimer, given the scope. The Gαs-HaloTag comparison, the V2R/AVP activation control, and the Gαs-ΔCt2 data collectively support functional assembly. One suggestion to strengthen the argument at no experimental cost: the authors have a strong internal control they do not use. If Gγ2-Halo were failing to incorporate with exogenous Gα12/Gα13, those conditions should converge on the Gnat value (~0.32 µm²/s); instead, they diverge sharply (0.14 and 0.20 µm²/s). This logic directly argues against the misassembly scenario and belongs in the Limitations section.

Promoter mismatch and subunit stoichiometry. Addressed as far as feasible. The reciprocal-promoter experiment in S6A is the appropriate control, and the limitation is now stated plainly.

Narrowing the conclusion/title. The new title and abstract largely address the scope.

The fixed-parameter reanalysis in S7 was needed and has been addressed. It is worth noting in the text that the fixed linking distance (0.7 µm) sits at the low end of the range used, which, if anything, biases against the faster groups.

The explicit statement at the end of the Statistical analysis section, the revised Fig. 3A legend, and the S2 Table resolve the data analysis issue.

The unsupported interpretive claim has been addressed by removing the sentence.

• However, the abstract overstates the comparison involving Gq. It states that Gα12/Gα13-containing heterotrimers show markedly reduced mobility "compared to those containing Gαi/o, Gαs, and Gαq." This is not what the statistics show: in Fig 3A, Gq is assigned BC and shares group C with G12 and G13, and in Fig 4B, Gq groups with G12 and G13 (B). The Results text is appropriately careful ("somewhat in between these groups"), but the abstract is not. Please either remove Gαq from that sentence or qualify it (e.g., "with Gαq intermediate").

• Residual overgeneralization in headings and the conclusion. The title and abstract were narrowed to Gβ1γ2-containing heterotrimers, but the Results heading ("Different families of G proteins exhibit distinct mobility patterns in live cells"), the closing sentence of the Discussion ("strong differences in mobility of different types of heterotrimeric G proteins"), and the phrase "in the broader context of G protein mobility" still generalize beyond the tested pairing. Please align these with the revised title.

• The single Gγ subtype is not listed as a limitation. Given that Gγ identity itself determines Gβγ membrane behavior, the restriction to Gβ1γ2 warrants a fourth item in the Limitations section, alongside expression level, density, and cell type.

• Statistical reporting is incomplete. The Methods define ε² and rank-biserial correlation, but no test statistics, p-values, or effect sizes appear in the Results or in a supporting table (only the regression output is reported). Please report these, state which test was applied to each figure (Kruskal-Wallis/Dunn vs Mann-Whitney), and specify the α and correction used for the compact letter display in Figs 3A and 4B.

• S6 and S7 carry a heavy interpretive burden for the descriptive data. S6A is cited in response to four separate reviewer concerns about expression levels, yet the legend states that no statistical inference was performed due to low sample size. This is understandable, but the wording throughout ("comparable mobility," "no significant effect") should be softened, since the absence of testing is not evidence of equivalence. Reporting n and the observed ranges for each S6 condition would help.

• Clarify the basis for the diffusion-state percentages. The values quoted in the Results (59/60/57% vs 44/41/41%) should be identified as pooled-track fractions or as means/medians of per-cell fractions, consistent with the new statement that cell-level medians are the statistical unit.

• Membrane density is reported as 0.14-0.20 molecules/µm² in the Results but as ~0.1-0.3 mol/um² in the TIRF Methods; please reconcile and use µm² consistently.

• "lipidation motive" is used instead of "lipidation motif" (Discussion).

• Inconsistent naming of the truncation mutant: "Gαs-ΔCt2" vs. "Gs-ΔCt2" (Methods, Limitations, S6 legend).

I think the manuscript is ready for publication after these edits.

**********

what does this mean?). If published, this will include your full peer review and any attached files.

If you choose “no”, your identity will remain anonymous but your review may still be made public.

**Do you want your identity to be public for this peer review?**  For information about this choice, including consent withdrawal, please see our Privacy Policy

Reviewer #1: No

Reviewer #2: No

Reviewer #3: **Yes:**  Evi Kostenis

Reviewer #4: No

**********

[NOTE: If reviewer comments were submitted as an attachment file, they will be attached to this email and accessible via the submission site. Please log into your account, locate the manuscript record, and check for the action link "View Attachments". If this link does not appear, there are no attachment files.]

To ensure your figures meet our technical requirements, please review our figure guidelines: https://journals.plos.org/plosone/s/figures

You may also use PLOS’s free figure tool, NAAS, to help you prepare publication quality figures: https://journals.plos.org/plosone/s/figures#loc-tools-for-figure-preparation.

NAAS will assess whether your figures meet our technical requirements by comparing each figure against our figure specifications.

Attachment Submitted filename: Reviewer Report - R1 - PONE-D-26-18579.docx

# Author comment: Author response to Decision Letter 2

**DOI:** 10.1371/journal.pone.0359304.r004

1 Sep 2026

Reviewer #1

1. The current limitations regarding the use of Gγ2-HaloTag as a reporter should be stated more explicitly. In particular, the authors cannot directly determine what fraction of labeled Gβ1γ2 is associated with the exogenously expressed Gα subtype, as opposed to endogenous Gα proteins or free Gβγ. I therefore recommend adding a clear statement to the Limitations section, for example: “We cannot directly determine the fraction of labeled Gβ1γ2 associated with the exogenously expressed Gα subtype versus endogenous Gα or free Gβγ.”

Response: We thank the reviewer for the suggestion. We have decided to directly state and address this possible caveat in the Limitations section of the manuscript. At the same time, we believe that the supplemental data from the Gs protein suggest that the mobilities reported by Gγ2-HaloTag are relevant indicators for individual G proteins (S6A-B Fig). Additionally, if we assume an issue in the assembly of G12 or G13, resulting in either endogenous Gα pairing or free Gβγ, the reported mobilities should shift towards the values observed in Gnat. Instead, we see that the values observed in G12 and G13 sharply diverge from those observed in Gnat (Fig 3A), indicating that the mobility difference between G12/13 and Gs, at the very least, cannot be explained by misassembly alone. (l.243-253)

2. For the experiments using Gα13-IRES-mCherry and Gαq-IRES-mCherry, the Methods should clearly state whether the mCherry signal was used as a criterion for cell selection. If it was used, the selection criteria should be described. If it was not used, this should also be clarified, particularly because the expression constructs for Gα13 and Gαq differ from those used for the other Gα subunits.

Response: Thank you for the suggestion. We have included a statement addressing the (non)usage of mCherry signal in the cell selection process in the manuscript. (l. 314-315)

3. In the Methods, the membrane density used for cell selection is described as approximately “0.1–0.3 mol/µm2.” This appears to be a unit error and should presumably be “0.1–0.3 molecules/µm2.” Please confirm and correct the unit accordingly.

Response: Thank you. This was a typographic error from earlier versions of the manuscript that went unnoticed. It has now been corrected. (l. 313)

Reviewer #4

The authors adequately validate that Gγ2-Halo reports the intended heterotrimer, given the scope. The Gαs-HaloTag comparison, the V2R/AVP activation control, and the Gαs-ΔCt2 data collectively support functional assembly. One suggestion to strengthen the argument at no experimental cost: the authors have a strong internal control they do not use. If Gγ2-Halo were failing to incorporate with exogenous Gα12/Gα13, those conditions should converge on the Gnat value (~0.32 µm²/s); instead, they diverge sharply (0.14 and 0.20 µm²/s). This logic directly argues against the misassembly scenario and belongs in the Limitations section.

Response: We highly appreciate the thoughtful suggestion. We decided to clearly articulate this point in the updated Limitations section. (l. 243-253)

It is worth noting in the text that the fixed linking distance (0.7 µm) sits at the low end of the range used, which, if anything, biases against the faster groups.

Response: Thank you for the comment. We included a statement regarding analysis parameters in the manuscript. (l. 338-340)

● However, the abstract overstates the comparison involving Gq. It states that Gα12/Gα13-containing heterotrimers show markedly reduced mobility "compared to those containing Gαi/o, Gαs, and Gαq." This is not what the statistics show: in Fig 3A, Gq is assigned BC and shares group C with G12 and G13, and in Fig 4B, Gq groups with G12 and G13 (B). The Results text is appropriately careful ("somewhat in between these groups"), but the abstract is not. Please either remove Gαq from that sentence or qualify it (e.g., "with Gαq intermediate").

Response: The comparison between Gq and the other subunits is indeed complicated, differing depending on the metric (fraction of diffusion states/overall diffusion coefficient/diffusion state-specific diffusion coefficient). The interpretation of the results in the abstract was too narrow and has been corrected. (l. 10)

● Residual overgeneralization in headings and the conclusion. The title and abstract were narrowed to Gβ1γ2-containing heterotrimers, but the Results heading ("Different families of G proteins exhibit distinct mobility patterns in live cells"), the closing sentence of the Discussion ("strong differences in mobility of different types of heterotrimeric G proteins"), and the phrase "in the broader context of G protein mobility" still generalize beyond the tested pairing. Please align these with the revised title.

Response: Thank you. The latter parts of the manuscript have been updated to reflect the adjusted characterization. (l. 85, 109, 168-169, 223)

● The single Gγ subtype is not listed as a limitation. Given that Gγ identity itself determines Gβγ membrane behavior, the restriction to Gβ1γ2 warrants a fourth item in the Limitations section, alongside expression level, density, and cell type.

Response: Thank you for the suggestion. We have decided to expand the Limitations chapter to better articulate this constraint in result interpretation. (l. 264-267)

● Statistical reporting is incomplete. The Methods define ε² and rank-biserial correlation, but no test statistics, p-values, or effect sizes appear in the Results or in a supporting table (only the regression output is reported). Please report these, state which test was applied to each figure (Kruskal-Wallis/Dunn vs Mann-Whitney), and specify the α and correction used for the compact letter display in Figs 3A and 4B.

Response: Thank you for the suggestion. The details of all statistical analyses are included in the supplementary table accompanying the publication, as mandated by PLOS ONE guidelines, which may not be available during the review process. At the same time, we agree that important statistical indicators should be included directly in the manuscript. To preserve readability, we have decided to include all the important statistical indicators in their corresponding figure legends. (l. 130-131, 155-156, 519-520, 531-532, 535-538, 540-545, 547-549, 552-555)

● S6 and S7 carry a heavy interpretive burden for the descriptive data. S6A is cited in response to four separate reviewer concerns about expression levels, yet the legend states that no statistical inference was performed due to low sample size. This is understandable, but the wording throughout ("comparable mobility," "no significant effect") should be softened, since the absence of testing is not evidence of equivalence. Reporting n and the observed ranges for each S6 condition would help.

Response: We thank the reviewer for the suggestion. Statistical analysis was not possible in S6A and S6B Fig due to the low effect size and the low sample size, respectively. The pairwise comparison in S6C Fig includes proper statistical analysis. We made sure to better explain this in the manuscript, as well as include statistical indicators, n, and observed ranges where applicable. (l. 535-538, 540-545, 547-549)

● Clarify the basis for the diffusion-state percentages. The values quoted in the Results (59/60/57% vs 44/41/41%) should be identified as pooled-track fractions or as means/medians of per-cell fractions, consistent with the new statement that cell-level medians are the statistical unit.

Response: Thank you. During the assessment of this part of the manuscript, we have noticed an oversight on our part, where in-text values of the mean per-cell fractions were not updated according to the analysis approach adjusted during the second round of review. We have made sure to correct this, as well as implement your suggestion in the manuscript. (l. 138-140, 162)

● Membrane density is reported as 0.14-0.20 molecules/µm² in the Results but as ~0.1-0.3 mol/um² in the TIRF Methods; please reconcile and use µm² consistently.

Response: The numerical discrepancy arises because the Results show the calculated average membrane density values of the different subunits, also shown in S1 Table, whereas the TIRF Methods report on the approximate acceptable range of values used for cell selection, within which almost all analyzed cells fall. The use of mol/um² in this instance was a typographical error and has been corrected. (l. 313)

● "lipidation motive" → "lipidation motif" (Discussion).

Response: Thank you. We adjusted the manuscript accordingly. (l. 190)

● Inconsistent naming of the truncation mutant: "Gαs-ΔCt2" vs. "Gs-ΔCt2" (Methods, Limitations, S6 legend).

Response: Thank you for the comment. We made sure to adjust the manuscript to correctly refer to the truncated mutant of the Gαs subunit as "Gαs-ΔCt2", and the resulting heterotrimer containing Gαs-ΔCt2 as "Gs-ΔCt2", consistent with other heterotrimer references in the manuscript. (l.238-239, 539)

I think the manuscript is ready for publication after these edits.

Attachment Submitted filename: Review response R2.docx

# Peer review: Decision Letter 2

**DOI:** 10.1371/journal.pone.0359304.r005

**Authors:** Yosuke Senju

13 Sep 2026

Heterotrimeric G proteins containing Gβ1γ2 subunits exhibit subtype-specific mobility differences in live cells

PONE-D-26-18579R2

Dear Dr. Bondar,

We’re pleased to inform you that your manuscript has been judged scientifically suitable for publication and will be formally accepted for publication once it meets all outstanding technical requirements.

Within one week, you’ll receive an e-mail detailing the required amendments. When these have been addressed, you’ll receive a formal acceptance letter and your manuscript will be scheduled for publication.

An invoice will be generated when your article is formally accepted. Please note, if your institution has a publishing partnership with PLOS and your article meets the relevant criteria, all or part of your publication costs will be covered. Please make sure your user information is up-to-date by logging into Editorial Manager at Editorial Manager® and clicking the ‘Update My Information' link at the top of the page. For questions related to billing, please contact billing support.

If your institution or institutions have a press office, please notify them about your upcoming paper to help maximize its impact. If they’ll be preparing press materials, please inform our press team as soon as possible -- no later than 48 hours after receiving the formal acceptance. Your manuscript will remain under strict press embargo until 2 pm Eastern Time on the date of publication. For more information, please contact onepress@plos.org.

Kind regards,

Yosuke Senju, Ph.D.

Academic Editor

PLOS One

Additional Editor Comments (optional):

Reviewers' comments:

Reviewer's Responses to Questions

**Comments to the Author**

Reviewer #4: All comments have been addressed

**********

2. Is the manuscript technically sound, and do the data support the conclusions?

Reviewer #4: Yes

**********

3. Has the statistical analysis been performed appropriately and rigorously?

Reviewer #4: Yes

**********

4. Have the authors made all data underlying the findings in their manuscript fully available?

The PLOS Data policy

Reviewer #4: Yes

**********

5. Is the manuscript presented in an intelligible fashion and written in standard English?

Reviewer #4: Yes

**********

Reviewer #4: The manuscript is ready for publication. The authors have addressed the comments adequately. No further comments from me.

**********

what does this mean?). If published, this will include your full peer review and any attached files.

If you choose “no”, your identity will remain anonymous but your review may still be made public.

**Do you want your identity to be public for this peer review?**  For information about this choice, including consent withdrawal, please see our Privacy Policy

Reviewer #4: No

**********

# Editor report: Acceptance letter

**DOI:** 10.1371/journal.pone.0359304.r006

**Authors:** Yosuke Senju

PONE-D-26-18579R2

PLOS One

Dear Dr. Bondar,

I'm pleased to inform you that your manuscript has been deemed suitable for publication in PLOS One. Congratulations! Your manuscript is now being handed over to our production team.

At this stage, our production department will prepare your paper for publication. This includes ensuring the following:

* All references, tables, and figures are properly cited

* All relevant supporting information is included in the manuscript submission,

* There are no issues that prevent the paper from being properly typeset

You will receive further instructions from the production team, including instructions on how to review your proof when it is ready. Please keep in mind that we are working through a large volume of accepted articles, so please give us a few days to review your paper and let you know the next and final steps.

Lastly, if your institution or institutions have a press office, please let them know about your upcoming paper now to help maximize its impact. If they'll be preparing press materials, please inform our press team within the next 48 hours. Your manuscript will remain under strict press embargo until 2 pm Eastern Time on the date of publication. For more information, please contact onepress@plos.org.

You will receive an invoice from PLOS for your publication fee after your manuscript has reached the completed accept phase. If you receive an email requesting payment before acceptance or for any other service, this may be a phishing scheme. Learn how to identify phishing emails and protect your accounts at https://explore.plos.org/phishing.

If we can help with anything else, please email us at customercare@plos.org.

Thank you for submitting your work to PLOS One and supporting open access.

Kind regards,

PLOS One Editorial Office Staff

on behalf of

Dr. Yosuke Senju

Academic Editor

PLOS One
